# Sodium Butyrate-Assisted Induction of Posterior Pre-Neural Progenitors from Pluripotent Stem Cells

**DOI:** 10.3390/ijms27146507

**Published:** 2026-07-22

**Authors:** Kyung Taek Oh, Deok Ho Kim, Wonjun Hong, Kyoungmin Park, Hakyoung You, Cheol-Koo Lee, Chulhong Oh, Gun-Hoo Park, Seungkwon You

**Affiliations:** 1 Laboratory of Cell Function Regulation, Department of Biotechnology, College of Life Sciences and Biotechnology, Korea University, Seoul 02841, Republic of Korea; ken2013@korea.ac.kr (K.T.O.); hwj4604@korea.ac.kr (W.H.); skskrudals@korea.ac.kr (K.P.); 2 Laboratory of Functional Genomics, Department of Biotechnology, College of Life Sciences and Biotechnology, Korea University, Seoul 02841, Republic of Korea; kdh0120@korea.ac.kr (D.H.K.); hkann_y@korea.ac.kr (H.Y.); cklee2005@korea.ac.kr (C.-K.L.); 3 Institute of Animal Molecular Biotechnology, Korea University, Seoul 02841, Republic of Korea; 4 Korea Institute of Ocean Science & Technology, 2670, Iljudong-ro, Gujwa-eup, Jeju-si 63349, Jeju-do, Republic of Korea; och0101@kiost.ac.kr (C.O.); gunhoopark@kiost.ac.kr (G.-H.P.)

**Keywords:** sodium butyrate, posterior neural development, pre-neural progenitors, ventral motor neuron development

## Abstract

Posterior axis development during mammalian embryogenesis is driven by transient progenitor states that give rise to neural and mesodermal lineages, including neuromesodermal progenitors (NMPs). In vitro derivation of posterior progenitor populations from human pluripotent stem cells (hPSCs) has relied on modulation of Wnt and FGF signaling; however, these approaches frequently generate heterogeneous and unstable cell populations. Here, we investigated whether sodium butyrate (NaB) supplementation could promote a posteriorly biased intermediate state without extensive extracellular signaling control. We show that NaB, a histone deacetylase inhibitor, promotes the induction of posterior pre-neural progenitors (PNPs) characterized by co-expression of CDX2 and SOX2, together with suppression of SOX1. Transcriptomic analyses revealed that NaB-treated cells exhibit a posteriorly enriched PNPs with restrained anterior neural differentiation, transient early *TBXT* induction, and progressive activation of posterior *HOX* genes, consistent with an incompletely caudalized intermediate rather than a fully specified NMP population. Importantly, these PNPs remained responsive to canonical neural tube patterning cues, including retinoic acid and smoothened agonists, enabling further differentiation toward ventral spinal cord lineages. Collectively, our findings demonstrate that NaB supplementation supports posterior PNPs from hPSCs, providing a simple and reproducible platform for modeling early posterior neural development in vitro.

## 1. Introduction

Axial elongation of the posterior body axis during mammalian embryogenesis is coordinated by transient progenitor populations that generate both neural and mesodermal derivatives [1,2]. Among these, neuromesodermal progenitors (NMPs) are a well-characterized bipotent population classically defined by the co-expression of SOX2 and T (Brachyury), together with the ability to contribute to the posterior neural tube and paraxial mesoderm [3,4]. During posterior neural development, posterior pre-neural progenitors (PNPs) arise as intermediate progenitor states in which posterior identity programs, including *CDX*-associated transcriptional features, are engaged while neural competence is retained prior to overt neural differentiation [5,6]. These transitional states provide a useful model for understanding how posterior neural progenitor identities are established and maintained.

Most in vitro approaches to derive posterior progenitor populations from human pluripotent stem cells (hPSCs) have focused on recapitulating Wnt- and FGF-dependent signaling environments [3,7,8,9]. Activation of these pathways can effectively induce posterior identity; however, establishing a well-defined PNP population with preserved neural competence remains challenging, as cells tend to transition toward more lineage-restricted neural or mesodermal programs. To improve differentiation efficiency and promote neural specification, additional pathway modulation strategies, including dual-SMAD inhibition, are often incorporated [8,10]. Despite these efforts, generating reproducible PNP populations remains difficult. In addition, these protocols typically involve complex combinations of cytokines and small molecules, which are highly sensitive to cell line-specific responses and culture conditions [9,11]. Consequently, batch-to-batch variability and limited reproducibility across experiments have been reported [12,13,14], suggesting that signaling-based approaches alone may not be sufficient to achieve stable posterior PNP induction.

Beyond extracellular signaling, epigenetic regulation has emerged as an important mechanism governing cell state transitions during development [15,16,17]. Sodium butyrate (NaB) is a well-established histone deacetylase (HDAC) inhibitor that has been widely used to influence stem cell fate and lineage specification [18,19,20,21,22]. In our previous study [23], NaB was included as one component of a small-molecule cocktail for the generation of induced neural stem cells (iNSCs) from human urine-derived cells. Under these conditions, a subset of the resulting iNSCs expressed posterior identity markers, including HOXB6 and HOXB8, suggesting the presence of a posterior PNP-like population with spinal cord characteristics. These observations prompted us to investigate whether NaB itself promotes the establishment of posterior PNP identity during pluripotent stem cell differentiation.

Based on these considerations, we hypothesized that supplementation of NaB in LSC medium, leukemia inhibitory factor (LIF), SB431542 (TGF-β inhibitor), and CHIR99021 (GSK3β inhibitor) could support the induction of a posterior PNP state characterized by concurrent CDX2 and SOX2 expression. Specifically, we postulated that NaB-containing conditions can promote posterior identity while preserving neural competence, thereby limiting mesodermal drift and premature lineage commitment during early differentiation. To address this hypothesis, we quantitatively compared the efficiency of CDX2^+^SOX2^+^ cell induction under NaB-supplemented, controlled conditions and examined temporal changes in transcriptional landscapes to assess the emergence of PNP features. Furthermore, we evaluated the functional competence by observing neural tube-like differentiation in response to retinoic acid (RA) and smoothened agonist (SAG). Through this integrated approach, we aimed to establish a simple and reproducible strategy for generating a CDX2^+^SOX2^+^ PNP population from hPSCs.

## 2. Results

### 2.1. Defined Culture Conditions Promote CDX2^+^SOX2^+^ Pre-Neural State

**Figure 1:**
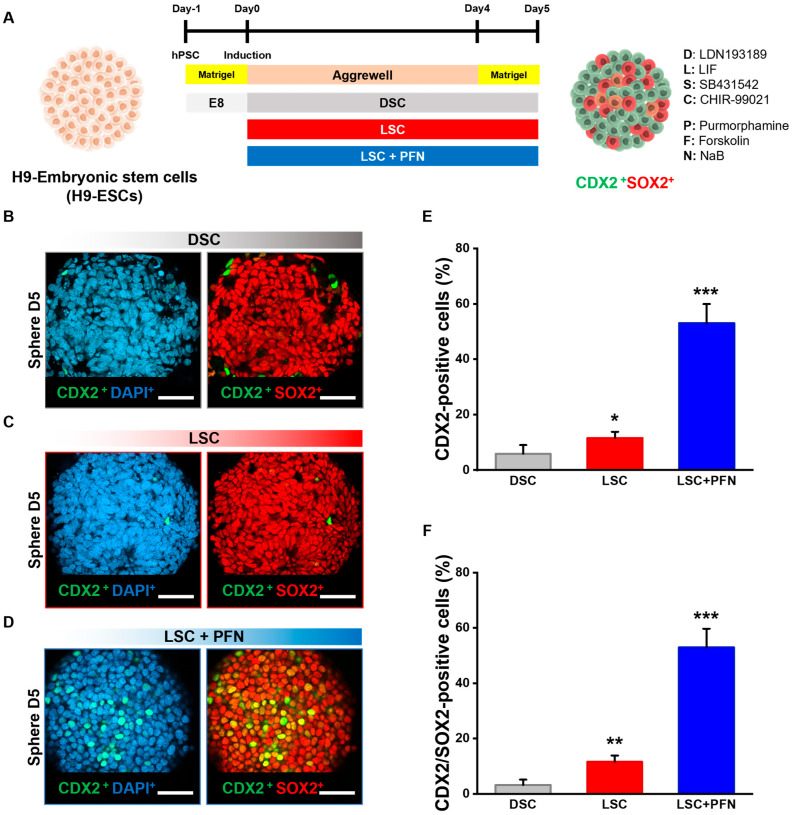
Induction of CDX2^+^SOX2^+^ pre-neural progenitors (PNPs) under defined differentiation conditions. (**A**) Schematic overview of the differentiation strategy used to evaluate the induction of CDX2^+^SOX2^+^ PNPs from H9 human embryonic stem cells (H9-ESCs). Cells were differentiated as three-dimensional spheres under three conditions: DSC, LSC, and LSC + PFN. (**B**–**D**) Representative confocal immunofluorescence images of spheres at day 5 of differentiation under DSC (**B**), LSC (**C**), and LSC + PFN (**D**) conditions. Cells were stained for CDX2 and SOX2, with nuclei counterstained with DAPI. Scale bars, 100 μm. (**E**,**F**) Quantification of CDX2-positive (**E**) and CDX2/SOX2-positive (**F**) cells expressed as percentages of total DAPI-positive cells. Quantification was performed using ImageJ (version 1.53k) based on confocal images obtained from independent differentiation experiments (*n* = 4). Data are presented as mean ± SD. Statistical significance was determined using one-way ANOVA followed by post hoc tests. * *p* < 0.05, ** *p* < 0.01, *** *p* < 0.001.

In our previous study [23], we observed that a subset of induced neural stem cells (iNSCs) expressed posterior identity markers. Based on the developmental association of these markers with posterior spinal cord identity, we hypothesized that the resulting iNSC population might contain posterior pre-neural progenitor (PNP)-like cells. To examine this possibility, we confirmed the presence of a CDX2^+^SOX2^+^ population within the iNSC colonies (Figure S1). This observation suggested that the combined culture condition used for iNSC induction can potentially induce a CDX2^+^SOX2^+^ intermediate state. Based on this finding, we investigated the earlier stages of differentiation through systematic comparison of defined signaling conditions. H9 human embryonic stem cells (H9-ESCs) were differentiated as three-dimensional spheres under three conditions: a differentiation signaling condition composed of LDN193189, SB431542, and CHIR99021 (DSC) [24]; a LIF-based condition of LSC [25]; and LSC supplemented with purmorphamine (P), forskolin (F), and NaB (N) (LSC + PFN) (Figure 1A). Five days post-differentiation, spheres were analyzed by immunofluorescence staining for CDX2 and SOX2 for the evaluation of their posterior pre-neural identity. Under DSC conditions, CDX2 expression was rarely detected, and CDX2^+^SOX2^+^ cells were largely absent (Figure 1B). There was some increase in CDX2-positive cells under LSC treatment; however, most cells were predominantly SOX2-positive, indicating limited induction of a CDX2^+^SOX2^+^ population (Figure 1C). In contrast, LSC + PFN conditions led to a significant increase in CDX2^+^SOX2^+^ cells throughout the spheres, consistent with enhanced induction of a posterior pre-neural identity (Figure 1D). Quantitative analysis confirmed that the proportion of CDX2-positive cells was significantly higher in the LSC + PFN group compared with DSC and LSC groups (Figure 1E). SOX2 expression was maintained at high levels in all treatment groups (Figure 1B–D), with only a modest but significant increase in CDX2^+^SOX2^+^ cells observed under LSC + PFN treatment (Figure 1F), which secured the posterior identity without loss of neural competence. Collectively, these results indicate that during early differentiation stages, there was a significant increase in CDX2^+^SOX2^+^ cells treated with LSC + PFN.

### 2.2. Stepwise Refinement of Culture Components Underlying Induction of a CDX2^+^SOX2^+^ Pre-Neural State

**Figure 2:**
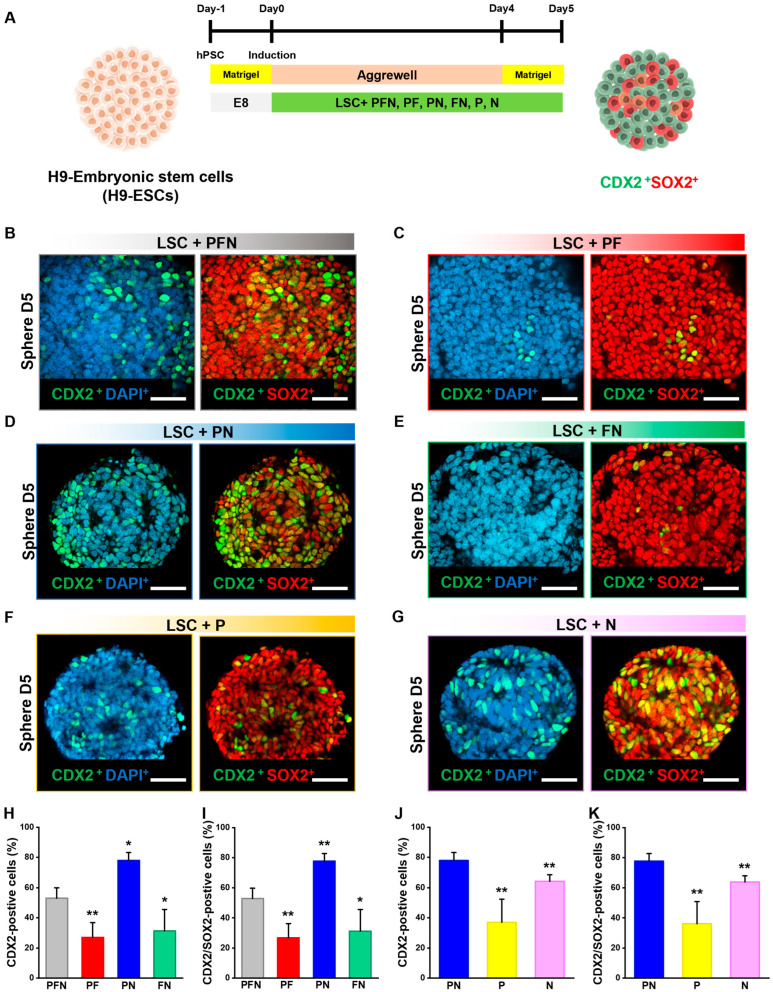
Stepwise refinement of culture components underlying induction of CDX2^+^SOX2^+^ PNPs. (**A**) Schematic overview of the stepwise refinement strategy used to dissect the contribution of PFN components under LSC background. H9-ESCs were differentiated as three-dimensional spheres in LSC supplemented with PFN or defined subsets of these components, including PF, PN, FN, P alone, or N alone. (**B**–**G**) Representative confocal immunofluorescence images of spheres at day 5 of differentiation cultured under LSC supplemented with PFN (**B**), PF (**C**), PN (**D**), FN (**E**), P alone (**F**), or N alone (**G**). Cells were stained for CDX2 and SOX2, with nuclei counterstained with DAPI. Scale bars, 100 μm. (**H**,**I**) Quantification of CDX2-positive (**H**) and CDX2/SOX2-positive (**I**) cells expressed as percentages of total DAPI-positive cells across PFN-derived combinations (PFN, PF, PN, and FN). (**J**,**K**) Quantification of CDX2-positive (**J**) and CDX2/SOX2-positive (**K**) cells expressed as percentages of total DAPI-positive cells following further refinement to individual components (P alone and N alone) under an LSC background. Quantification was performed using ImageJ based on confocal images obtained from independent differentiation experiments (*n* = 4). Data are presented as mean ± SD. Statistical significance was determined using one-way ANOVA followed by appropriate post hoc tests. * *p* < 0.05, ** *p* < 0.01.

To characterize the individual components of PFN combination during the induction of the CDX2^+^SOX2^+^ pre-neural state with progenitor characteristics from iNSCs, we specified the culture components under LSC background (Figure 2A). H9-ESCs were differentiated into three-dimensional spheres in LSC supplemented with PFN or with defined subsets of the PFN components, and marker expression was assessed at day 5 by immunofluorescence analysis. Consistent with our previous observation, LSC + PFN conditions enhanced CDX2^+^SOX2^+^ cells throughout the spheres (Figure 2B). The proportion of CDX2-positive cells was markedly reduced, but SOX2 expression was preserved when N was excluded (LSC + PF) (Figure 2C). In contrast, LSC supplemented with P and N (LSC + PN) maintained a high proportion of CDX2^+^SOX2^+^ cells in comparison to LSC + PFN (Figure 2D). The combination of F and N (LSC + FN) supported only a moderate level of CDX2 expression, indicating that the components of PFN contribute individually to posterior identity induction (Figure 2E). Quantitative analysis confirmed that CDX2-positive cell percentages were significantly higher under LSC + PN conditions than under LSC + PF or LSC + FN conditions, while SOX2 positivity remained high across all PFN-derived combinations (Figure 2H,I). While some variation in spheroid size was observed between conditions, this may arise from variability in initial cell aggregation within microwells during spheroid formation. Importantly, the differences in fluorescence signals primarily reflect changes in marker expression rather than size-dependent effects. These observations identified PN as a refined combination, sufficient to support CDX2 expression in the context of LSC-based differentiation.

To further refine the culture components supporting this pre-neural state, we next evaluated the individual contributions of P and N. Under LSC supplemented with P (LSC + P), CDX2-positive cells were detected at relatively low frequencies (Figure 2F). In contrast, LSC supplemented with N (LSC + N) resulted in a significantly higher proportion of CDX2-positive cells while maintaining SOX2 expression (Figure 2G). Quantitative analysis was consistent with these observations, with a greater portion of CDX2-positive cells under LSC + N compared with LSC + P, with a slight difference in SOX2-positive cell percentages (Figure 2J,K). Together, these results indicate that NaB-containing LSC conditions are sufficient to maintain posterior identity without compromising neural marker expression.

### 2.3. Dose-Dependent Effects of NaB Defining an Optimal Window for PNP Induction

**Figure 3:**
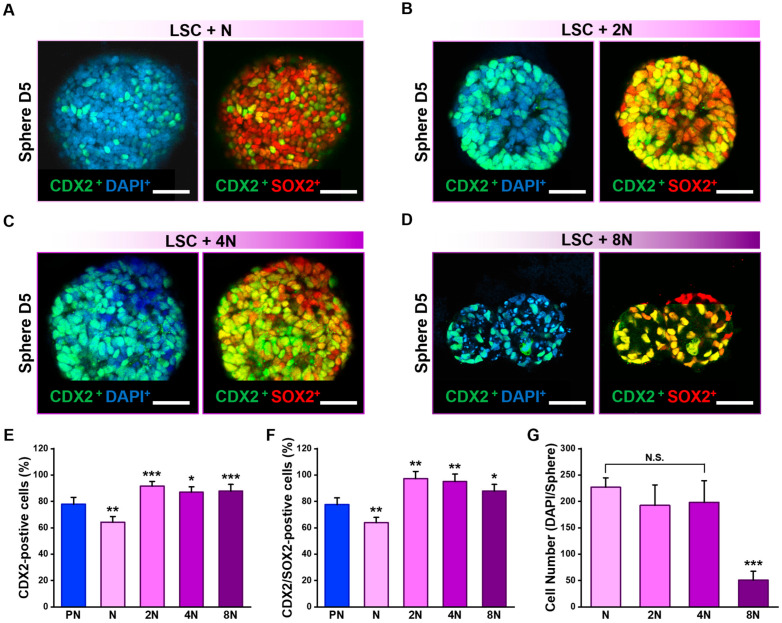
Dose-dependent refinement of NaB identifies an optimal window for generating CDX2^+^SOX2^+^ PNPs. (**A**–**D**) Representative confocal immunofluorescence images of H9-ESC-derived spheres at day 5 of differentiation cultured under an LSC background supplemented with increasing concentrations of NaB: N (**A**), 2N (**B**), 4N (**C**), and 8N (**D**). Cells were stained for CDX2 and SOX2, with nuclei counterstained with DAPI. Scale bars, 100 μm. (**E**,**F**) Quantification of CDX2-positive (**E**) and CDX2/SOX2-positive (**F**) cells expressed as percentages of total DAPI-positive cells across NaB dose conditions. PN condition is included as a reference condition from component screening experiments. (**G**) Quantification of total cell number per sphere, calculated based on DAPI-positive nuclei, across NaB dose conditions. Quantification was performed using ImageJ based on confocal images obtained from independent differentiation experiments. Data are presented as mean ± SD. Statistical significance was determined using one-way ANOVA followed by post hoc tests. * *p* < 0.05, ** *p* < 0.01, *** *p* < 0.001; N.S., not significant.

Previously, we identified NaB as a key component capable of inducing PNP under LSC conditions, accompanied by the emergence of a CDX2^+^SOX2^+^ pre-neural state. We next examined whether this effect was dependent on the concentration of NaB. H9-ESCs were differentiated as three-dimensional spheres with LSC containing increasing concentrations of NaB, referred to as N (100 μM), 2N, 4N, and 8N conditions, with numerical prefixes indicating fold increases relative to the N condition; analyses were performed at day 5 by immunofluorescence staining (Figure 3A–D). Under LSC + N conditions, spheres exhibited a substantial population of CDX2^+^SOX2^+^ cells (Figure 3A). Increasing the NaB concentration to 2N and 4N further enhanced the proportion of CDX2-positive cells while maintaining robust SOX2 expression, indicating a dose-dependent reinforcement of posterior pre-neural identity within this range (Figure 3B,C). Quantitative analysis confirmed a significant increase in CDX2-positive cell percentages under 2N and 4N conditions compared with N, whereas SOX2-positive cell proportions remained consistently high across these conditions (Figure 3E,F). The PN condition, included as a control condition consistent with earlier experiments, was used for comparison. In contrast, the 8N condition resulted in a marked reduction in sphere size and cellular density, as shown in the significant decrease in the number of DAPI-positive cells per sphere (Figure 3D,G). Although the remaining cells were both positive for CDX2 and SOX2, the substantial decline in total cell number under the 8N condition suggests reduced cellular fitness, viability and proliferative capacity. Therefore, this condition was excluded in subsequent analyses. Although both 2N and 4N conditions enhanced CDX2 expression without a detectable reduction in SOX2 positivity, the 2N condition was selected for subsequent experiments, as it efficiently induced CDX2^+^SOX2^+^ progenitor while maintaining overall cell number.

### 2.4. Molecular Characterization of LSC + 2N-Derived Cells Revealing a Posterior Biased Pre-Neural Features

**Figure 4:**
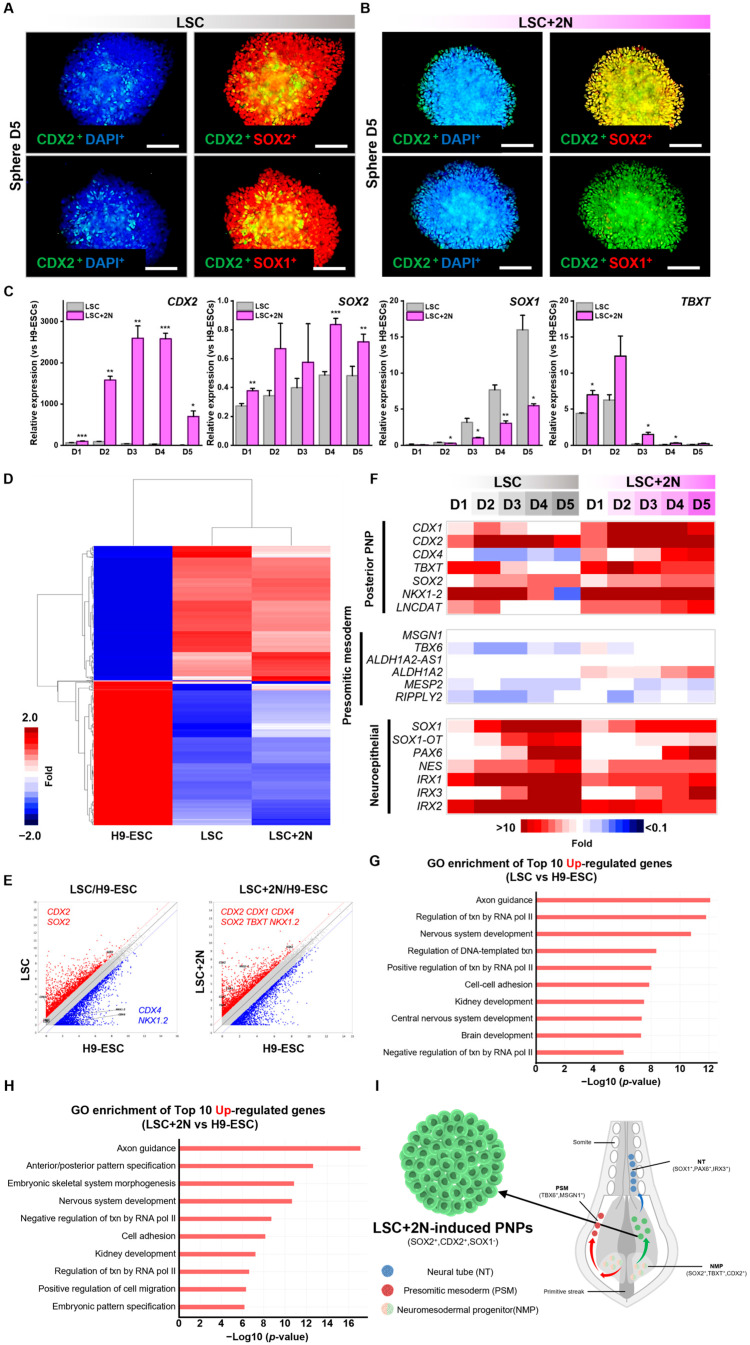
Molecular characterization of CDX2^+^SOX2^+^ PNPs promoted under LSC + 2N conditions. (**A**,**B**) Representative immunofluorescence images of H9-ESC-derived spheres at day 5 of differentiation cultured under LSC (**A**) or LSC + 2N (**B**). Cells were stained for CDX2 and SOX2 (**upper panels**) or CDX2 and SOX1 (**lower panels**), with nuclei counterstained with DAPI. Scale bars, 200 μm. (**C**) Quantitative PCR analysis of lineage-associated gene expression across differentiation days 1–5 in LSC and LSC + 2N cultures. Relative expression levels of *CDX2*, *SOX2*, *SOX1*, and *TBXT* are shown and normalized to H9-ESCs. (**D**) Hierarchical clustering heatmap of differentially expressed genes comparing H9-ESC, LSC D5, and LSC + 2N D5 (z-score-normalized expression). (**E**) Scatter plots of gene expression in H9-ESC vs. LSC or LSC + 2N. Smaller black labels indicate PNP-associated marker genes included in the analysis. Among these, genes highlighted in red represent upregulated genes, whereas those in blue indicate downregulated genes. (**F**) Heatmap of selected marker genes across D1–D5 under LSC and LSC + 2N conditions, including posterior PNP-associated genes, PSM-associated genes, and neural differentiation (Neuroepithelial)-associated genes (fold change relative to H9-ESC; color scale as shown). (**G**,**H**) GO Biological Process enrichment showing the top 10 significantly enriched biological process terms identified from the set of upregulated genes in LSC vs. H9-ESC (**G**) and LSC + 2N vs. H9-ESC (**H**), plotted as −log10 (*p* value). (**I**) Schematic summary illustrating the proposed molecular identity of LSC + 2N-derived cells as a posteriorly biased pre-neural population exhibiting PNP-like characteristics. Arrows indicate the representative cell populations corresponding to each colored label: green, pre-neural progenitor (PNP); blue, neural tube (NT); red, presomitic mesoderm (PSM); and light green and light red half, neuromesodermal progenitor (NMP). Data are presented as mean ± SD. Statistical significance was determined using two-way ANOVA followed by post hoc tests. * *p* < 0.05, ** *p* < 0.01, *** *p* < 0.001.

For the molecular characterization of the CDX2^+^SOX2^+^ population induced under LSC and LSC + 2N conditions, we first performed immunofluorescence analyses of day 5 spheroids. Under both conditions, SOX2 expression was broadly maintained throughout the spheroids; however, the spatial distribution and co-expression patterns of CDX2 and SOX1 differed between conditions. In LSC spheroids, the expression of CDX2 was not uniformly distributed as SOX2, and SOX1 expression was prominently detected. In contrast, LSC + 2N conditions exhibited sustained and widespread co-expression of CDX2 and SOX2 with comparatively reduced SOX1, consistent with a PNP state, rather than an overt neuroepithelial identity (Figure 4A,B). Temporal differences between conditions are evident at earlier differentiation stages (days 1–4). Under the LSC condition, CDX2 expression was diminutive at day 1 and became apparent from day 2 in a subset of cells, whereas under LSC + 2N, CDX2 expression was noted from day 1, which was more apparent from day 2 onward. In parallel, SOX1 expression increased from day 2 under LSC, while it remained consistently low under LSC + 2N (Figure S3). To quantify temporal changes in lineage-associated gene expression, qPCR analysis was performed from days 1 to 5. LSC + 2N cultures showed early induction and sustained elevation of *CDX2* expression relative to LSC cultures. *SOX2* expression was maintained under both conditions, whereas *SOX1* expression was significantly reduced in LSC + 2N cultures, particularly at later time points, consistent with restrained anterior neural differentiation. In addition, the mesoderm-associated marker TBXT was transiently upregulated at early time points under LSC + 2N conditions and subsequently declined. This transient expression pattern is consistent with the activation of an early posterior axial progenitor-like program prior to the establishment of a pre-neural identity, rather than sustained commitment to mesodermal fates (Figure 4C).

To assess transcriptome-wide differences, we performed hierarchical clustering and pairwise comparisons using RNA-seq profiles. Unsupervised clustering separated H9-ESC from day 5 LSC and LSC + 2N samples, indicating a differentiation-driven transcriptional shift, while the LSC and LSC + 2N samples were grouped more closely with each other than with H9-ESC (Figure 4D). Pairwise scatter plot analyses further revealed extensive transcriptome remodeling under both treatment groups. Notably, LSC + 2N cultures showed more pronounced induction of posterior PNP-associated markers including *CDX1/2/4*, *TBXT*, and *NKX1-2*, while maintaining high SOX2 expression. In contrast, LSC cultures exhibited weaker or absent induction of several of these markers (e.g., *NKX1-2* and *CDX4*), despite upregulated *SOX2*. (Figure 4E). Consistent with this posteriorly biased yet pre-neural transcriptional state, analysis of lineage-associated markers showed that LSC + 2N cultures maintained elevated *SOX2* expression while exhibiting reduced expression of genes associated with neuroepithelial or neural differentiation, including *SOX1*, *SOX1-OT*, *PAX6*, *NES*, and IRX family genes (*IRX1*, *IRX2*, and *IRX3*), relative to LSC cultures. (Figure 4F). Markers associated with presomitic mesodermal progenitor genes (*TBX6*, *MSGN1*, *MESP2*) remained low under both conditions, supporting the interpretation that LSC + 2N conditions support a posterior pre-neural state rather than promoting progression toward terminal neural or mesodermal differentiation. In parallel, both conditions showed progressive induction of posterior HOX transcripts over time, consistent with a gradual shift toward caudal identity during early differentiation. Importantly, LSC + 2N cultures revealed earlier onset and higher expression levels of caudal HOX genes than LSC cultures (Figure S8A), suggesting that NaB-based HDAC inhibition may facilitate HOX activation, potentially through increased chromatin accessibility at HOX regulatory regions, rather than imposing a single fixed transcriptional program [26].

Gene ontology (GO) enrichment analysis provided an orthogonal overview of the transcriptional differences between LSC and LSC + 2N conditions. GO Biological Process terms enriched among genes upregulated in LSC + 2N cultures were primarily associated with embryonic patterning and anterior–posterior (A–P) specification, consistent with the posterior pre-neural identity. In contrast, genes upregulated under LSC conditions were enriched for broader neurodevelopmental programs, including brain and central nervous system development, cilium assembly, and axon guidance, indicating a relative shift toward a more canonical neural differentiation-associated signature (Figure 4G,H). Downregulated GO terms were similar in both conditions, which were associated with migration- and adhesion-related processes, as well as apoptosis or protein catabolic pathways (Figure S8B,C). Collectively, these data indicate that LSC + 2N induces a CDX2 and SOX2 co-expressed transcriptional state, posterior positional identity, while restraining progression toward terminal neural or mesodermal lineages. Based on these molecular features, LSC + 2N-derived cells exhibit a posteriorly biased PNP characterized by distinct molecular features [5] (Figure 4I).

### 2.5. Induction of CDX2^+^SOX2^+^ PNPs Across Multiple Human Pluripotent Stem Cell Lines

**Figure 5:**
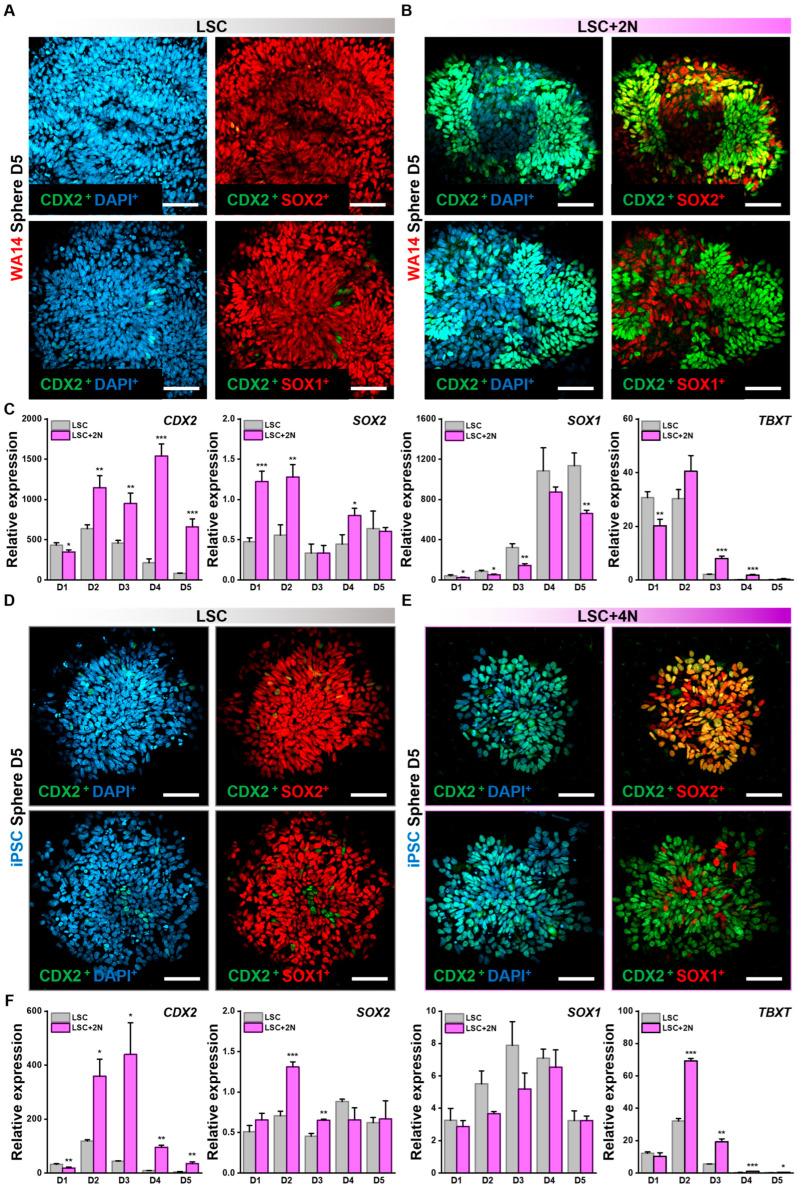
Induction of CDX2^+^SOX2^+^ PNPs across multiple hPSC lines. (**A**,**B**) Representative confocal immunofluorescence images of WA14-ESC-derived spheres at day 5 of differentiation cultured under LSC (**A**) or LSC + 2N (**B**). Cells were stained for CDX2 and SOX2 (**upper panels**) or CDX2 and SOX1 (**lower panels**), with nuclei counterstained with DAPI. Scale bars, 100 μm. (**C**) Quantitative PCR analysis of lineage-associated gene expression across differentiation days 1–5 in WA14-ESC cultures under LSC and LSC + 2N conditions. Relative expression levels of *CDX2*, *SOX2*, *SOX1*, and *TBXT* are shown and normalized to undifferentiated WA14-ESCs. (**D**,**E**) Representative confocal immunofluorescence images of induced pluripotent stem cell (iPSC)-derived spheres at day 5 of differentiation cultured under LSC (**D**) or LSC + 4N (**E**). Cells were stained for CDX2 and SOX2 (upper panels) or CDX2 and SOX1 (**lower panels**), with nuclei counterstained with DAPI. Scale bars, 100 μm. (**F**) Quantitative PCR analysis of lineage-associated gene expression across differentiation days 1–5 in iPSC cultures under LSC and LSC + 4N conditions. Relative expression levels of CDX2, SOX2, SOX1, and TBXT are shown and normalized to undifferentiated iPSCs. Data are presented as mean ± SD. Statistical significance was determined using two-way ANOVA followed by post hoc tests. * *p* < 0.05, ** *p* < 0.01, *** *p* < 0.001.

The induction of CDX2^+^SOX2^+^ PNPs under LSC-based conditions was next evaluated across multiple human pluripotent stem cell (hPSC) lines. The sphere cultures of another human embryonic stem cell line, WA14-ESCs, were analyzed at day 1–5 by immunofluorescence and quantitative gene expression analysis. In WA14-ESCs, LSC + 2N conditions resulted in a significant increase in CDX2^+^SOX2^+^ cells compared with LSC, validating the molecular and phenotypic features observed in H9-ESCs (Figure 5A,B, Figures S4 and S7A). SOX1 expression remained comparatively lower under LSC + 2N conditions, consistent with maintenance of a PNP state rather than progression toward definitive neural differentiation. Temporal gene expression analyses further supported sustained *CDX2* expression and preserved *SOX2* levels under LSC + 2N conditions, accompanied by reduced *SOX1* expression and transient early induction of *TBXT* (Figure 5C).

We next examined whether a similar PNP state could be established in induced pluripotent stem cells (iPSCs). In this setting, LSC supplemented with a higher NaB concentration (LSC + 4N) was used (Figure S5). Under these conditions, iPSC-derived spheres exhibited clear co-expression of CDX2 and SOX2, while SOX1 expression remained relatively restrained compared with LSC controls (Figure 5D,E, Figures S6 and S7B). Quantitative PCR analyses across differentiation days 1–5 revealed elevated *CDX2* expression and maintained *SOX2* levels during early differentiation, alongside transient upregulation of *TBXT* followed by a decline at later stages, consistent in both PSC-derived cultures (Figure 5F). Collectively, these results validate the induction of a CDX2^+^SOX2^+^ PNP under NaB supplemented LSC-based conditions in multiple hPSC lines, including both embryonic and induced pluripotent stem cells.

### 2.6. Receptivity of LSC + NaB-Derived PNP to Canonical Neural Tube Patterning Cues

**Figure 6:**
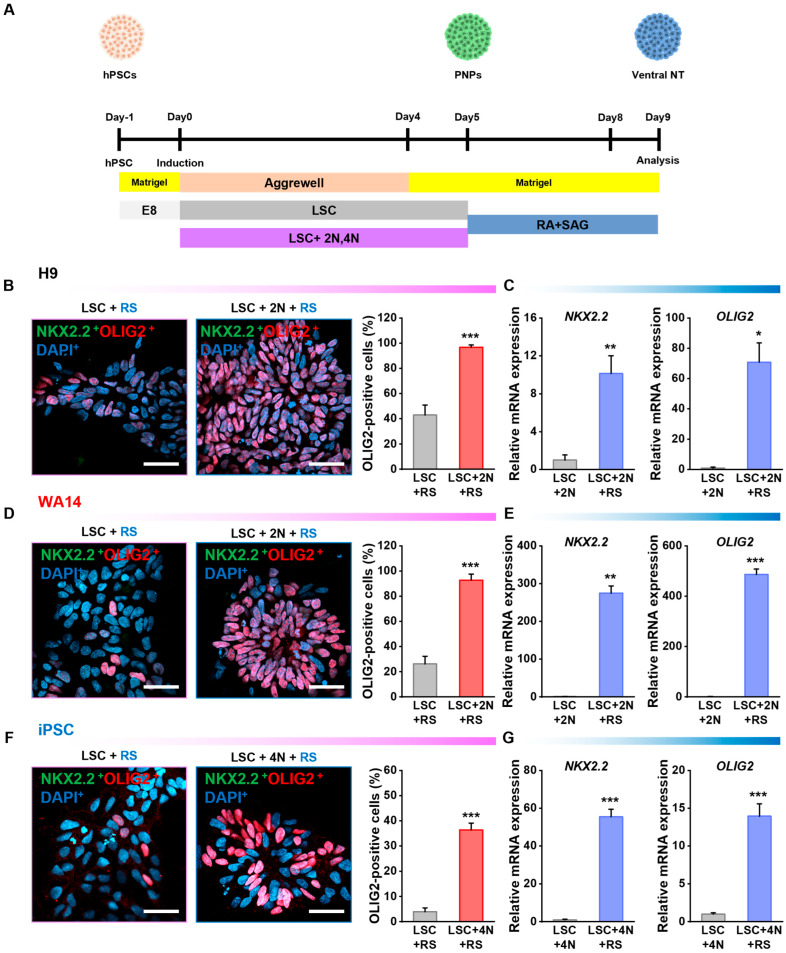
LSC + NaB-derived PNPs respond to canonical neural tube patterning cues. (**A**) Schematic overview of the patterning strategy. CDX2^+^SOX2^+^ PNPs were generated from hPSCs under LSC + 2N or LSC + 4N until day 5, followed by addition of RA and SAG (RS) from day 5 to day 9 to induce ventral neural tube patterning. (**B**,**C**) Representative confocal immunofluorescence images and quantification of OLIG2^+^ cells. (**B**) and qPCR analysis (**C**) showing induction of ventral neural tube markers NKX2.2 and OLIG2 in H9-ESC-derived cultures following RA + SAG treatment, compared with untreated controls. Nuclei were counterstained with DAPI. Scale bars, 100 μm. (**D**,**E**) Representative confocal immunofluorescence images and quantification of OLIG2^+^ cells (**D**) and qPCR analysis (**E**) demonstrating RA + SAG-induced expression of NKX2.2 and OLIG2 in WA14-ESC-derived cultures under LSC + 2N conditions. (**F**,**G**) Representative confocal immunofluorescence images and quantification of OLIG2^+^ cells (**F**) and qPCR analysis (**G**) showing induction of NKX2.2 and OLIG2 in iPSC-derived cultures differentiated under LSC supplemented with 4N sodium butyrate (LSC + 4N) following RA + SAG treatment. Quantitative PCR data are presented as mean ± SD from independent experiments (*n* = 4). Statistical significance was determined using one-way or two-way ANOVA followed by appropriate post hoc tests. * *p* < 0.05, ** *p* < 0.01, *** *p* < 0.001.

To assess whether the PNPs generated under LSC + NaB conditions retain competence to respond to developmental patterning cues, LSC and LSC + NaB conditioned cells were given cues for ventral neural tube patterning using RA and SAG (Figure 6A). Patterning was initiated on day 5 and evaluated on day 9 across H9-ESCs, WA14-ESCs, and iPSCs. In H9-ESC-derived cultures, LSC cells treated with RA + SAG (RS) showed minimal expression of ventral neural tube markers. In contrast, LSC + 2N induced PNPs with RS treatment; robust induction of *NKX2.2* and *OLIG2* was observed by qPCR, with OLIG2 showing clear protein-level induction as confirmed by immunofluorescence and qPCR analysis (Figure 6B,C). Similar responses were observed in WA14-ESC-derived cultures treated with LSC + 2N + RS, showing marked upregulation of NKX2.2 and OLIG2 compared with LSC controls (Figure 6D,E). We also examined whether PNPs derived from iPSCs exhibited similar patterning competence. Under LSC + 4N conditions, iPSC-derived CDX2^+^SOX2^+^ cells responded robustly to RS exposure, showing transcriptional induction of *NKX2.2* and *OLIG2*, accompanied by pronounced OLIG2 expression at the protein level (Figure 6F,G). Immunocytochemistry further showed that HOXC9 was expressed in a high proportion of cells across H9, WA14, and iPSC lines (Figure S9). Together, these results indicate that PNPs induced under LSC + NaB conditions remain broadly responsive to caudal–ventral neural tube patterning signals.

### 2.7. Characterization of LSC + 2N-Derived PNPs with Progression Toward Caudal Patterning and Motor Neuron Differentiation

**Figure 7:**
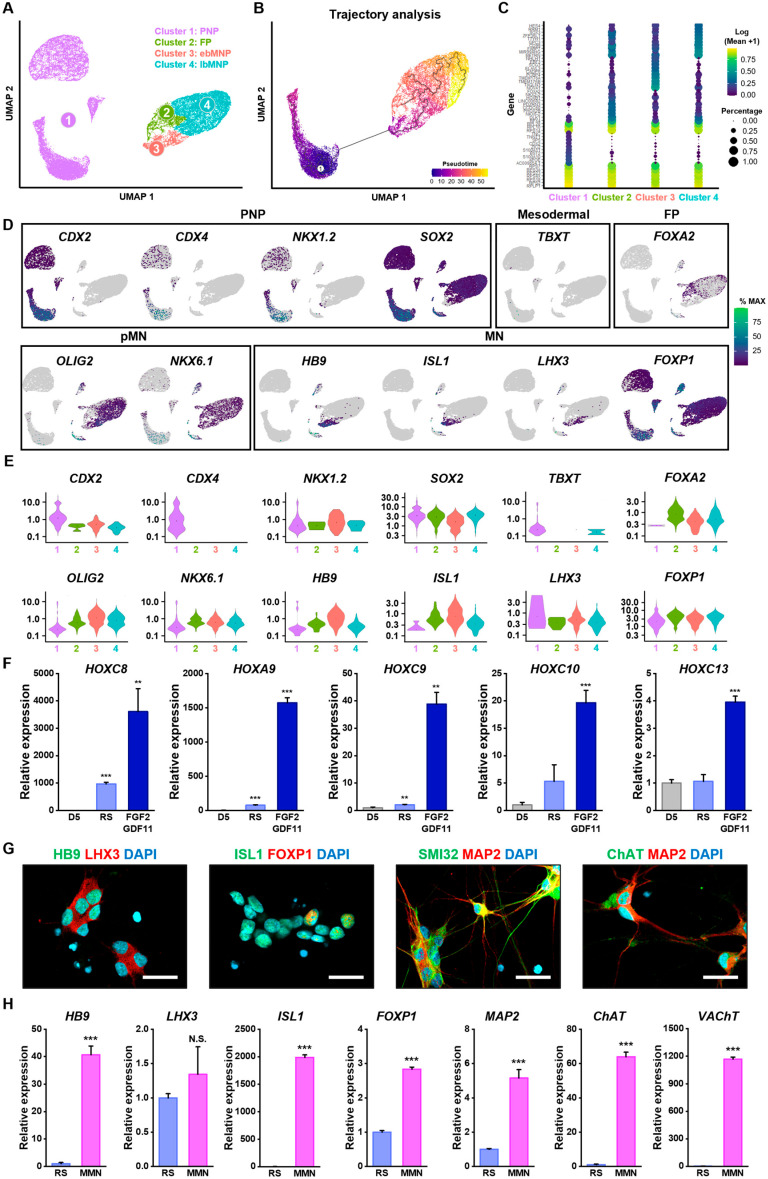
Integrated single-cell and marker-based comparison of RA + SAG (RS) and LSC + 2N conditions during spinal cord lineage induction and motor neuron differentiation. (**A**) UMAP-based clustering of single-cell transcriptomes. UMAP analysis was performed to resolve transcriptionally distinct cell populations. Cells were grouped into four clusters corresponding to PNP, floor plate (FP), early-born MNP, and late-born MNP, based on unsupervised clustering. (**B**) Trajectory inference was conducted to reconstruct the developmental continuum among the identified clusters. Pseudotime ordering revealed a directional transition from posterior neural progenitor states toward ventral spinal cord progenitor and motor neuron-related lineages, indicating progressive differentiation along a common developmental trajectory. (**C**) Differential expression analysis identified cluster-specific transcriptional signatures. The resulting gene expression profiles highlight molecular distinctions among posterior neural progenitors and ventral spinal cord progenitor subpopulations, supporting the separation of clusters observed in the UMAP embedding. (**D**) Plots showing the expression patterns of representative markers grouped by functional categories, including posterior identity-associated markers (*CDX2*, *CDX4*), pre-neural marker (*NKX1.2*), neural progenitor marker (*SOX2*), mesoderm-associated marker (*TBXT*) and pMN-associated markers (*OLIG2*, *NKX6.1*), floor plate marker (*FOXA2*), and motor neuron markers (*HB9*, *ISL1*, *LHX3*, and *FOXP1*). Category labels are indicated directly on the figure. (**E**) Violin plots summarizing marker expression across clusters. (**F**) RT–qPCR analysis of *HOX* gene expression (*HOXC8*, *HOXA9*, *HOXC9*, *HOXC10*, and *HOXC13*) in the indicated conditions (LSC + 2N (D5), RS, and FGF2 + GDF11). (**G**) Representative immunofluorescence images of differentiated motor neuron cultures stained for HB9/LHX3, ISL1/FOXP1, SMI32/MAP2, and CHAT/MAP2 (with DAPI). Scale bars, 100 μm. (**H**) RT–qPCR analysis of lineage-specific markers following terminal differentiation. Marker expression was evaluated for HB9 and LHX3 (medial motor column; MMC), ISL1 (hypaxial motor column; HMC and preganglionic motor column; PGC), FOXP1 (preganglionic motor column; PGC), SMI32 and MAP2 (mature neuronal markers), and ChAT and VAChT (motor neuron markers). The MMN group represents mature motor neuron markers. Data are presented as mean ± SD from independent experiments (*n* = 4). Statistical significance was determined using one-way ANOVA followed by appropriate post hoc tests. ** *p* < 0.01, *** *p* < 0.001. N.S., not significant.

Single-cell RNA sequencing was used to compare early differentiation outcomes between RS and LSC + 2N conditions. UMAP visualization showed that cells segregated by condition, with RS and LSC + 2N forming distinct transcriptional states rather than a single continuous manifold (Figure S10A). Unsupervised clustering showed multiple discrete subpopulations (clusters 1–4) across the combined dataset (Figure 7A), indicating heterogeneity associated with PNP and ventral motor neuron progenitor (pMN)-related identities under these patterning conditions. Marker expression analyses further demonstrated differential enrichment of PNP, ventral progenitor, and early motor neuron-associated genes across individual clusters (Figure S10B,C), which support functional stratification of the identified populations. Pseudotime analysis suggested a progression from the LSC + 2N-associated state toward the RS-associated state along an ordered trajectory, consistent with stepwise differentiation dynamics observed during development (Figure 7B). In addition, differential expression analyses identified cluster-specific gene expression signatures, which indicated the molecular distinction among clusters 1–4 (Figure 7C).

The molecular identities of these subpopulations were analyzed by canonical neural markers, caudal pre-neural markers and ventral spinal cord markers on the UMAP embedding (Figure 7D) and across clusters (Figure 7E). LSC + 2N enhanced the expression levels of posterior identity-associated markers (*CDX2/4*) and the pre-neural marker *NKX1.2*, a characteristic marker of caudal PNP [5,27]. *SOX2* remained broadly expressed across clusters, consistent with a progenitor-like baseline shared among subsets. In parallel, pMN-related transcription factors (*OLIG2*, *NKX6.1*) [14] were detected in specific clusters, and markers defining motor neuron identity (HB9, ISL1, LHX3, and FOXP1) were also observed. In addition, the floor plate marker FOXA2 was detected, whereas the mesoderm-associated marker TBXT was not prominently expressed. Collectively, these single-cell analyses indicate that LSC + 2N conditions bias early differentiation toward a posterior PNP state, whereas RS conditions favor ventral pMN-associated transcriptional programs (Figure 7D,E).

To further assess rostro–caudal patterning competence, we evaluated posterior *HOX* gene expression following exposure to additional patterning cues. FGF2 and GDF11, which have been implicated in posterior axial patterning and late *HOX* activation [5,28,29], induced increased expression of posterior HOX genes, including *HOXC8*, *HOXA9*, *HOXC9*, *HOXC10*, and *HOXC13*, compared with unpatterned controls (Figure 7F). These observations indicate that LSC + 2N-derived PNPs remain responsive to cues that promote posterior *HOX* gene activation.

Finally, patterned cultures gave rise to differentiated thoracic motor neuron subtypes expressing markers consistent with ventral spinal cord lineages, including *HB9* and *LHX3* (Medial Motor Column; MMC), *ISL1* (Hypaxial Motor Column; HMC and preganglonic motor column; PGC), and *FOXP1* (PGC motor neuron subtype), as well as *SMI32*, *MAP2*, *ChAT*, and *VAChT* following further maturation (Figure 7G,H) [30]. Collectively, these results demonstrate that CDX2^+^SOX2^+^ PNPs induced under LSC + NaB conditions retain competence to respond to canonical rostro–caudal and dorso–ventral patterning cues, supporting their patterning competence and developmental potential.

## 3. Discussion

The initial observation of CDX2 and SOX2 co-expression in induced neural stem cell (iNSC) cultures, despite the absence of intentional posterior patterning, suggested that specific combinations of signaling regulation, growth factor support, and Sodium butyrate (NaB) supplementation can create a permissive environment for the establishment and maintenance of a CDX2^+^SOX2^+^ intermediate state (Figure S1). This finding implies that posterior pre-neural features may arise not only through direct posteriorizing cues [3,5,31] but also through culture contexts that balance differentiation-promoting signals with factors that preserve cellular competence [25]. Comparison between DSC and LSC conditions highlighted the importance of this balance (Figure 1B,C,E,F). Standard dual-SMAD inhibition is primarily optimized for neuroepithelial induction and does not promote CDX2-expressing posterior states [30]. Although LIF has been employed in the original protocol to support anterior neuroepithelial differentiation [25], our study showed that its inclusion only partially preserved posterior features while maintaining SOX2, and that the supplementation of PFN is required to increase the proportion of CDX2^+^SOX2^+^ cells (Figure 1D–F). These observations are in accordance with the emerging concepts in developmental biology suggesting that posterior pre-neural progenitor (PNP) identities can be supported by permissive environments rather than instructive lineage-specifying signals alone [32]. Similar principles have been proposed in studies of neural priming and axial progenitors, where modulation of signaling balance alters the temporal persistence of transient developmental states [16,27,28,33]. From this perspective, the CDX2^+^SOX2^+^ population can potentially represent a posterior pre-neural intermediate that is preferentially induced and maintained under NaB-containing conditions.

Systematic partition of PFN components with LSC background identified NaB as a key contributor in posterior PNP induction (Figure 2A). Removal of NaB led to a pronounced reduction in CDX2-positive cells, whereas the presence of NaB preserved robust CDX2^+^SOX2^+^ populations with maintained SOX2 expression (Figure 2C–K). Further investigation of the individual components demonstrated that NaB alone was sufficient to maintain a substantial proportion of CDX2^+^SOX2^+^ cells, whereas purmorphamine alone was not effective (Figure 2F,G). Overall, these results indicate that NaB supplementation, rather than additional morphogen-related signaling, is a key factor promoting posterior pre-neural identity. Given the well-established HDAC inhibitory activity of NaB, its reported epigenetic properties may contribute to this process [15,22,34]. However, the underlying chromatin-level mechanisms were not directly investigated in this study. In this context, NaB may facilitate a cellular environment that maintains neural competence and posterior transcriptional programs rather than acting as a posteriorizing signal [35,36,37,38]. Accordingly, the present study does not establish NaB as a unique PNP induction factor but rather as a setting in which posterior identity can be maintained while preserving neural marker expression.

The dose-dependent effect of NaB can be further utilized to refine PNP induction conditions. Increasing NaB concentrations from N to 2N and 4N enhanced CDX2 expression while preserving SOX2 positivity. However, higher concentrations of NaB (8N) displayed a reduction in sphere size and total cell numbers despite retention of pre-neural markers (Figure 3A–G). The separation between marker expression and cellular yield may suggest that high levels of HDAC inhibition may impose cellular stress that limits proliferative capacity or cell survival, rather than altering lineage-associated transcriptional identity [34,38]. Consistent with the previous studies in PSCs and early progenitors, such dose-dependent trade-offs between lineage specification and cellular fitness can be associated with impaired cell cycle progression, metabolic stress, and activation of stress-related transcriptional responses [39]. Collectively, these findings indicate the optimal levels of NaB for the induction of a CDX2^+^SOX2^+^ PNP.

Induction of the CDX2^+^SOX2^+^ population under LSC + 2N conditions was in alignment with a posteriorly biased pre-neural transcriptional state (Figure 4A–F). Sustained co-expression of CDX2 and SOX2, together with reduced SOX1 expression, is consistent with a PNP state in which posterior identity is maintained while overt neural differentiation is restrained (Figure 4A–C) [1,27,29,40]. Consistent with this interpretation, HES5 expression remained low under LSC + 2N conditions but was markedly upregulated following RS-mediated ventral neural tube patterning (Figure S11). Previous developmental studies have shown that HES5 expression becomes prominent during neuroepithelial and neural tube specification rather than during the earlier pre-neural progenitor stage [41], supporting the interpretation that the LSC + 2N-derived population represents a posterior pre-neural intermediate rather than a specified neural tube identity.

At the same time, the pattern of *HOX* gene expression indicates incomplete caudalization. Although select trunk-associated posterior *HOX* genes were induced over time (Figure S8A), full posterior axial identity is typically associated with broader engagement of *HOX* clusters, including *HOXC* genes [7,42,43,44]. Accordingly, the observed *HOX* expression profile is consistent with a posteriorly biased PNP state, and not with a fully caudalized axial progenitor, which is also supported by global transcriptome analyses. Furthermore, LSC + 2N-derived PNPs remained responsive to subsequent posteriorizing signals, including FGF2 and GDF11, resulting in increased sacral HOX gene expression (Figure 7F). This developmental responsiveness further supports their identity as posterior PNP intermediates.

Hierarchical clustering showed that both day 5 differentiation conditions were clearly distinguished from H9-ESC, evident by substantial differentiation-associated transcriptional remodeling, while LSC and LSC + 2N samples clustered closely but exhibited distinct gene expression differences (Figure 4D). Consistent with this separation, scatter plot comparisons indicated that posterior and pre-neural-associated transcripts, including *CDX2, NKX1-2*, and *CDX4*, were more strongly upregulated in the LSC + 2N condition than in the LSC condition (Figure 4E). Neural competence was preserved under LSC + 2N conditions; persistent *SOX2* expression and attenuation of anterior neural differentiation and neural maturation were apparent in *SOX1*, *PAX6*, *NES*, and *IRX* family genes (Figure 4F). Moreover, GO enrichment analyses revealed increased anterior–posterior pattern specification and broader embryonic patterning processes in the LSC + 2N condition, which was consistent with the posterior-biased PNP state (Figure 4G,H). This transcriptional configuration is consistent with delayed neural commitment and maintenance of a developmentally flexible PNP [3,16,45]. Transient upregulation of *TBXT* at early differentiation stages, followed by its subsequent decline, suggests activation of a posterior PNP program without sustained mesodermal commitment (Figure 4C) [46,47,48]. Consistent with increased CDX2 expression, posterior HOX gene expression was observed (Figure S8), supporting the induction of posterior identity under LSC + 2N conditions. Taken together, these molecular features support the interpretation that LSC + 2N-derived cells represent a posterior PNP population, rather than a bona fide neuromesodermal progenitor state (Figure 4F). Accordingly, the PNP identified in this study represents a posteriorly biased intermediate that retains responsiveness to neural patterning cues without acquiring full neuromesodermal identity.

Extension of the differentiation conditions to multiple hPSC lines demonstrates that induction of a CDX2^+^SOX2^+^ PNP is reproducible across embryonic and induced pluripotent stem cells (Figure 5 and Figure S7), supporting the broader applicability of NaB supplementation in pluripotent stem cell differentiation. Notably, iPSC-derived cultures required a higher NaB concentration (4N) to achieve CDX2^+^SOX2^+^ cells than in ESCs. Such differences are consistent with known variability in epigenetic landscapes among PSC lines, particularly in iPSCs, where residual epigenetic memory and reprogramming-associated heterogeneity can influence chromatin accessibility and responsiveness to epigenetic modulators [49,50]. These findings suggest that the optimal concentration of NaB may vary depending on the intrinsic properties of individual PSC lines. Importantly, despite the difference in NaB sensitivity, the molecular features of the induced PNP were largely conserved across different cell lines. NaB supplementation provides a practical advantage, as its concentration can be optimized across different PSC lines without altering the overall differentiation protocol. In this context, future studies may utilize NaB in diverse PSC lines and genetic backgrounds to facilitate the generation and maintenance of other transient developmental intermediates.

In addition to NaB supplementation, the use of three-dimensional (3D) spheroid culture may have contributed to the establishment of the posterior pre-neural state observed in this study. While adherent culture systems have been widely used to efficiently generate NMP-like or PNP populations, the 3D configuration provides a distinct microenvironment that enhances cell–cell interactions and spatial organization. These features may support the establishment of posterior identity while limiting premature neural differentiation, as reflected by sustained CDX2 and SOX2 expression together with reduced SOX1 induction. In this context, the 3D culture system may facilitate the establishment of a pre-neural intermediate state rather than promoting rapid lineage commitment. This interpretation is further supported by comparative analysis between adherent and spheroid conditions (Figure 4 and Figure S2).

Furthermore, cluster 2 showed co-expression of *OLIG2* and *NKX2.2* (Figure 7), markers that define adjacent ventral progenitor domains (pMN vs. p3) and are known to sharpen the pMN–p3 boundary through cross-repressive interactions [51]. The differing protein levels of NKX2.2 and OLIG2 may likely reflect differences in expression timing or protein stability during early patterning, rather than reflecting distinct underlying transcriptional states. This co-expression pattern was also observed in clusters 2–4, consistent with the progenitor states that emerge during gliogenic transition-related stages within oligodendrocyte-lineage contexts under RS conditions [52]. A recent study identified an early OLIG2^+^NKX2-2^+^ progenitor population as ventral progenitors of motor neurons (vpMNs) and a major source for FOXP1^+^ LMC-like motor neurons in a human-specific context [53]. However, a subset of FOXP1^+^ motor neurons in our system arose during RS-mediated ventral motor neuron differentiation with thoracic identity. This observation is consistent with the possibility that RS-based patterning conditions trajectories related to PGC-like differentiation, rather than a single motor neuron subtype. In addition, we also observed clusters that were more prevalent for the canonical OLIG2^+^/NKX2-2^−^ profile, suggesting the coexistence of distinct ventral progenitor states within the patterned cultures. Further studies should be executed to determine whether additional patterning cues or temporal modulation can bias differentiation toward alternative thoracic motor neuron subtype columns (MMC/HMC).

The present study does not delineate the epigenetic mechanisms through which NaB promotes the establishment of the CDX2^+^SOX2^+^ PNP state. Although NaB is widely used as a histone deacetylase inhibitor [20,21], Western blot analysis at day 5 revealed only a modest, non-significant increase in global H3K9 acetylation under LSC + 2N conditions (Figure S12). Because this bulk endpoint analysis cannot resolve transient changes occurring at earlier stages or locus-specific acetylation at posterior lineage-associated regulatory regions, the present findings do not provide direct evidence of chromatin remodeling or targeted epigenetic regulation [54,55]. Direct evidence of chromatin remodeling or locus-specific regulatory changes was not examined in this work, and targeted investigation should be performed in future studies [56]. In addition, our data support that NaB facilitates PNP identity; however, whether this cellular state can be maintained during prolonged culture remains to be determined [28].

Nonetheless, our findings establish a simple and reproducible differentiation condition for inducing a CDX2^+^SOX2^+^ PNP from hPSCs while preserving neural competence (Figure 6), without reliance on complex extracellular signaling modulation. NaB supplementation represents the central experimental strategy of this study for inducing transient developmental intermediates, and here we present a simple and experimentally accessible condition for in vitro posterior neural development. Building upon this strategy, future studies may evaluate whether similar NaB-based approaches can be extended to other developmentally related lineages.

## 4. Materials and Methods

### 4.1. Cells

This study was approved by the Institutional Review Board at the Korea University (IRB approval number: KUIRB-2025-0070-01). H9 (WA09) and H14 (WA14) human embryonic stem cells (hESCs), provided by WiCell Research Institute (Madison, WI, USA) (WiCell Research Institute, Madison, WI, USA), CMC-003-iPSCs [57] were maintained in E8 medium (Gibco, Brooklyn, NY, USA) on Matrigel (BD Biosciences Clontech, Palo Alto, CA, USA) coated cell culture dishes. Cells were cultured at 37 °C with 5% CO_2_, and the medium was changed daily. For passaging, cells were dissociated using Versene (Gibco) and replated at a ratio of approximately 1:6–1:10, depending on confluency. During passaging, 10 μM Y-27632 (ROCK inhibitor; Tocris Bioscience, Bristol, UK) was added for the first 24 h to enhance cell survival. Cells were typically passaged every 4–5 days upon reaching ~80% confluency. H9-ESC, WA14-ESC and iPSC were collectively referred to as hPSCs in this paper.

### 4.2. Cell Culture and Differentiation

For the differentiation of pre-neural progenitor (PNP) cells, human pluripotent stem cells (hPSCs) were seeded into AggreWell™400 plates (Stemcell Technologies, Kent, WA, USA) and cultured in LSC + 2N medium. The composition of the basal medium was a 1:1 mixture of DMEM/F12 and Neurobasal (Thermo Fisher Scientific, Waltham, MA, USA), 0.5× N2, 0.5× B27 (Thermo Fisher Scientific), 1× penicillin/streptomycin, 1× L-glutamine, and 1× non-essential amino acids (Thermo Fisher Scientific). The basal medium supplemented with 10 ng/mL recombinant human LIF (MilliporeSigma, Burlington, MA, USA), 2 μM SB431542 (Tocris Bioscience, Bristol, UK) and 2.5 μM CHIR99021 (Tocris Bioscience) is referred to as the LSC medium. The addition of small molecules, including 64 μg/mL vitamin C (MilliporeSigma) and 200 μM NaB (Tocris Bioscience), is referred to as LSC + 2N medium, which was used as the final differentiation condition for PNP induction.

During optimization experiments, additional small molecules—0.5 μM purmorphamine (Tocris Bioscience), 10 μM forskolin (Tocris Bioscience), and 100 nM LDN193189 (Tocris Bioscience)—were tested to evaluate their potential effects on differentiation efficiency. However, these components were not included in the final LSC + 2N differentiation protocol.

After 4 days post-induction, spheres were transferred onto Matrigel-coated plates and maintained in LSC + 2N medium. For the differentiation of motor neuron progenitor (MNP) cells, PNP cells were cultured in basal medium supplemented with 0.1 μM retinoic acid (Sigma-Aldrich, St. Louis, MO, USA) and 1 μM SAG (MedChemExpress, Monmouth Junction, NJ, USA) (MNP medium). Three days post-transfer, colonies were subcultured using versene (Gibco) and seeded onto Matrigel-coated plates in MNP medium. For motor neuron generation, MNPs were cultured in basal medium supplemented with 0.5 μM retinoic acid, 0.2 μM SAG, and 64 μg/mL Vitamin C, and cells were cultured for 4 days. After 4 days, MNPs were dissociated with Accutase (Sigma, Cape Town, South Africa) and seeded onto poly-L-ornithine (Thermofisher Scientific)/laminin (sigma)-coated plates in maturation medium consisting of basal medium, 10 μM cAMP (Tocris Bioscience), 64 μg/mL Vitamin C, 10 ng/mL BDNF (PeproTech, Rocky Hill, NJ, USA), and 10 ng/mL GDNF (PeproTech) for 15 days.

### 4.3. RT-PCR and qRT-PCR

Total RNA was extracted from cultured cells using TRIzol reagent (Invitrogen, Carlsbad, CA, USA) according to the manufacturer’s instructions. Complementary DNA (cDNA) was generated with AccuPower^®^ RT PreMix (Bioneer, Daejeon, Republic of Korea) using oligo(dT)18 primers (Bioneer). Gene-specific amplification was carried out with designated primer sets, and GAPDH served as the internal reference gene for normalization. Quantitative real-time PCR was conducted using iQ SYBR Green Supermix (Bio-Rad, Hercules, CA, USA). Detailed sequences are provided in Supplementary Table S3.

### 4.4. Immunocytochemistry and Quantification

Cells were fixed with 4% paraformaldehyde for 20 min at room temperature. For permeabilization and blocking, samples were treated with PBS supplemented with 0.3% Triton X-100 and 5% donkey serum for 20 min at room temperature. Primary antibodies were diluted in PBS containing 5% donkey serum and applied overnight at 4 °C. After washing with PBS, cells were incubated with the appropriate secondary antibodies for 1 h at room temperature, followed by nuclear counterstaining with DAPI for 5 min. Cells were rinsed twice with PBS between each step. Fluorescent images were acquired using a fluorescence microscope (IX71, Olympus, Tokyo, Japan) or a confocal laser scanning microscope (LSM900, Carl Zeiss, Oberkochen, Germany), and confocal images represent single optical sections. A complete list of antibodies is provided in Supplementary Tables S1 and S2. Quantification of immunofluorescence images was performed using ImageJ (NIH, version 1.53k). CDX2^+^, SOX2^+^, and double-positive cells were manually counted and expressed as a percentage of total DAPI^+^ nuclei. At least three independent fields were analyzed per condition across independent experiments. All quantifications were performed in a blinded manner.

### 4.5. RNA Isolation

Total RNA was extracted either with TRIzol Reagent or the Maxwell^®^ RSC miRNA Kit (Promega™ Corporation, Madison, WI, USA) in accordance with the manufacturers’ protocols. RNA integrity was evaluated using the Agilent 4200 TapeStation system (Agilent Technologies, Amstelveen, The Netherlands). RNA concentration was measured with a Qubit 4 or Qubit Flex fluorometer (Thermo Fisher Scientific, Wilmington, DE, USA).

### 4.6. Total RNA-Seq Library Preparation and Sequencing

rRNAs were removed using the RiboCop rRNA Depletion Kit (LEXOGEN, Inc., Vienna, Austria). Libraries were prepared from rRNA-depleted total RNA samples using the CORALL RNA-Seq V2 Library Prep Kit (LEXOGEN, Inc., Austria). The rRNA-depleted RNAs were also used for cDNA synthesis and shearing, following the manufacturer’s instructions. Indexing was performed using the Lexogen UDI 12-nt Unique Dual Indexing V2. The enrichment step was carried out using PCR. Subsequently, libraries were checked using the TapeStation 4200 System (Agilent Technologies, Amstelveen, The Netherlands) or the Agilent 2100 Bioanalyzer (Agilent Technologies, Amstelveen, The Netherlands) to evaluate the mean fragment size. Quantification was performed using qPCR with the KAPA library quantification kit (Kapa Biosystems, Wilmington, MA, USA) according to the manufacturer’s library quantification protocol. High-throughput sequencing was performed as paired-end 100 bp sequencing using the NovaSeq 6000 (Illumina, Inc., San Diego, CA, USA).

### 4.7. Total RNA-Seq Data Analysis

Quality control of raw sequencing data was performed using FastQC (version 0.12.0) [58]. Adapter and low-quality reads were removed using fastp (version 1.0.0) [59]. The trimmed reads were mapped to the reference genome using STAR (version 2.7.11a) [60]. Read quantification was processed using Salmon (version 1.10.0) [61]. The read counts were processed based on the TMM + CPM normalization method using the Python “conorm” package (version 1.2.0) [62]. Data mining and graphical visualization were performed using ExDEGA (version 5.2.1) (Ebiogen Inc., Seoul, Republic of Korea).

Gene ontology (GO) analysis was performed using DAVID. Differentially expressed genes (DEGs) were selected based on an adjusted *p*-value (false discovery rate, FDR) < 0.05 and log_2_ fold change > 1. GO enrichment analysis was performed using all upregulated genes, and the top 10 enriched biological process terms were selected based on statistical significance. Mitochondrial and ribosomal genes were excluded from the analysis to avoid bias associated with housekeeping functions.

### 4.8. sc-RNA-seq Library Preparation and Sequencing

Single-cell suspensions were washed and resuspended in 0.04% BSA in PBS. Cells were counted with Countess^TM^ III (Thermo Fisher, Waltham, MA, USA) for concentration determination. Single-cell RNA-sequencing libraries were prepared using the Chromium GEM-X Single Cell Gene Expression v4 Kit (10X Genomics, Pleasanton, CA, USA) in accordance with the manufacturer’s protocol. Cells were diluted into the Chromium GEM-X Single Cell 3’ Chip to yield a recovery of approximately 10,000 single cells. Following the library preparation, the libraries were sequenced in multiplex on the NovaSeq X Plus sequencer (Illumina, San Diego, CA, USA) to produce an average of at least 30,000 reads per single cell.

### 4.9. sc-RNA-seq Data Analysis

Sequencing reads were processed with Cell Ranger version 9.0.0 (10X Genomics, Pleasanton, CA, USA) using the human reference transcriptome GRCh38 provided by 10x Genomics. From the gene expression matrix, the downstream analyses were carried out using R version 4.2.2. Quality control, filtering, data clustering and visualization, and the differential expression analyses were performed using the WinSeurat program (Ebiogen Inc., Seoul, Republic of Korea) based on the Seurat version 4.2.2 R package [63] with some custom modifications to the standard pipeline. For each individual dataset, genes expressed in cells with <500 UMIs and <300 genes were removed from the gene expression matrix. Single cells with >10% UMIs mapped to mitochondrial genes and cells with a complexity score <0.8 were both removed. After the SCTransform normalization, the expression of each gene was scaled by regressing out the number of UMIs in each cell. PCA on the gene expression matrix was performed, and the first 20 principal components were used for clustering and visualization. Clustering was performed with a resolution of 0.5, and visualization was performed using UMAP (Uniform Manifold Approximation and Projection). For trajectory inference and cluster-specific differential expression analyses, Monocle3 was used to identify genes distinguishing individual clusters and to infer pseudotemporal ordering of cells.

### 4.10. Statistical Analysis

All quantitative results are presented as the mean ± standard deviation (SD) from a minimum of three independent replicates. Statistical analyses were conducted using unpaired two-tailed Student’s *t*-tests or analysis of variance (ANOVA), as appropriate. Statistical significance was defined as * *p* < 0.05, ** *p* < 0.01 and *** *p* < 0.001.

## 5. Conclusions

In this study, we describe that sodium butyrate (NaB) supplementation under LSC-based conditions enables the induction of a CDX2^+^SOX2^+^ pre-neural progenitor (PNP) from human pluripotent stem cells (hPSCs). Systematic refinement of culture components and dose optimization identified NaB as a key factor that is capable of reinforcing posterior pre-neural identity while preserving neural competence. Molecular analyses indicate that the induced state is PNP, characterized by posterior bias and delayed neural commitment, and is in the incomplete caudalized pre-neural intermediate state. Importantly, PNPs generated under LSC + NaB conditions are responsive to canonical neural tube patterning cues, supporting functional competence and developmental responsiveness. The reproducibility of this strategy across multiple hPSC lines highlights the broad applicability of NaB supplementation for posterior PNP induction across diverse genetic backgrounds. Collectively, our findings establish a simple and reproducible differentiation condition that supports the generation of a CDX2^+^SOX2^+^ posterior pre-neural population from hPSCs. This approach provides a practical in vitro model for posterior neural development and a foundation for further investigation of the molecular mechanisms regulating posterior PNP specification.

## Data Availability

The data presented in this study are available on request from the corresponding author due to ongoing research.

## Supplementary Materials

The following supporting information can be downloaded at: https://www.mdpi.com/article/10.3390/ijms27146507/s1.

## Abbreviations

The following abbreviations are used in this manuscript:

NMPs	Neuromesodermal progenitors
PNPs	Pre-neural progenitors
NaB	Sodium butyrate
