# Supplementary material for: Sodium Butyrate-Assisted Induction of Posterior Pre-Neural Progenitors from Pluripotent Stem Cells
Source: Int J Mol Sci. 2026 Jul 22;27(14):6507. doi: 10.3390/ijms27146507 (PMC13409835; doi:10.3390/ijms27146507)
Supplement: Supplementary file 1 [file ijms-27-06507-s001.zip › ijms-4397317-supplementary.pdf]

Sodium Butyrate-Assisted Induction of Posterior Pre-Neural Progenitors from Pluripotent Stem Cells

Kyung Taek Oh [1], Deok Ho Kim [2], Wonjun Hong [1], Kyoungmin Park [1,3], Hakyoung You [2], Cheol-Koo Lee [2,3], Chulhong Oh [4], Gun-Hoo Park [4] and Seungkwon You [1,3,*]

[1] Laboratory of Cell Function Regulation, Department of Biotechnology, College of Life Sciences and
Biotechnology, Korea University, Seoul 02841, Republic of Korea; ken2013@korea.ac.kr (K.T.O.); hwj4604@korea.ac.kr (W.H.); skskrudals@korea.ac.kr (K.P.)

[2] Laboratory of Functional Genomics, Department of Biotechnology, College of Life Sciences and
Biotechnology, Korea University, Seoul 02841, Republic of Korea; kdh0120@korea.ac.kr (D.H.K.); hkann_y@korea.ac.kr (H.Y.); cklee2005@korea.ac.kr (C.-K.L.)

[3] Institute of Animal Molecular Biotechnology, Korea University, Seoul 02841, Republic of Korea

[4] Korea Institute of Ocean Science & Technology, 2670, Iljudong-ro, Gujwa-eup, Jeju-do, Jeju-si 63349, Republic of Korea; och0101@kiost.ac.kr (C.O.); gunhoopark@kiost.ac.kr (G.-H.P.)

* Correspondence: bioseung@korea.ac.kr; Tel.: +82-2-3290-3057; Fax: +82-2-3290-3507

Supplementary Methods

Western blotting

To assess overall H3K9 acetylation during posterior PNP induction, H9-ESCs were differentiated under LSC or LSC+2N conditions and harvested on day 5. Total protein was extracted using RIPA buffer (Thermo Fisher Scientific) supplemented with protease inhibitors, followed by centrifugation at 12,000 × g for 30 min at 4 °C. Protein concentrations were measured using the Bradford assay (Bio-Rad, Hercules, CA, USA). Equal amounts of protein were separated on 4–12% SDS–PAGE gels (Invitrogen) and transferred to polyvinylidene difluoride membranes. The membranes were blocked with 5% skim milk in Tris-buffered saline containing 0.1% Tween 20 (Sigma-Aldrich) and incubated overnight at 4 °C with antibodies against H3K9ac (Cell Signaling Technology, #9671; 1:1000) and total histone H3 (Upstate, #06-755; 1:1000). After washing, the membranes were incubated with horseradish peroxidase-conjugated anti-rabbit secondary antibodies (1:1000) at room temperature. Protein bands were visualized using an enhanced chemiluminescence detection system (ECL kit; Pierce, Rockford, IL, USA) and quantified using ImageJ software. H3K9ac levels were normalized to total histone H3.

Supplementary Table S1. List of Primary Antibodies.

Primary antibody	Dilution	Host	Provider
SOX2	1:200	Goat	R&D systems(AF2018)
SOX1	1:200	Goat	R&D systems(AF3369)
CDX2	1:200	Mouse	Biogenex(MU392A-5UC)
HOXC9	1:100	Mouse	Abcam(ab50839)
OLIG2	1:200	Rabbit	IBL (IBL-18953)
NKX2.2	1:200	Mouse	DSHB (74.5A5)
MNX1	1:200	Mouse	DSHB(81.5C10)
ISL1	1:200	Mouse	DSHB(40.2D6)
LHX3	1:1000	Rabbit	Proteintech(20745-1-AP)
FOXP1	1:200	Goat	R&D systems(AF4534)
MAP2	1:1000	Rabbit	Sigma-aldrich(AB5622)
CHAT	1:200	Goat	Sigma-aldrich(AB144P)

Supplementary Table S2. List of Secondary Antibodies.

Secondary antibody	Dilution	Host	Provider
Alexa Fluor 488 (Mouse IgG)	1:500	Donkey	Thermo Fisher (A-21202)
Alexa Fluor 488 (Rabbit IgG)	1:500	Donkey	Thermo Fisher (A-21206)
Alexa Fluor 488 (Goat IgG)	1:500	Donkey	Thermo Fisher (A-11055)
Alexa Fluor 488 (Mouse IgM)	1:500	Goat	Thermo Fisher (A-21042)
Alexa Fluor 488 (Rat IgG)	1:500	Donkey	Thermo Fisher (A-21208)
Alexa Fluor 594 (Mouse IgG)	1:500	Donkey	Thermo Fisher (A-21203)
Alexa Fluor 594 (Rabbit IgG)	1:500	Donkey	Thermo Fisher (A-21207)
Alexa Fluor 594 (Goat IgG)	1:500	Donkey	Thermo Fisher (A-11058)
Alexa Fluor 594 (Rat IgG)	1:500	Donkey	Thermo Fisher (A-21209)

Supplementary Table S3. List of Primer sequences

Gene	Primer	Sequence (5’-3’)
TBXT	Forward	AGGTACCCAACCCTGAGGA
	Reverse	GCAGGTGAGTTGTCAGAATAGGT
CDX2	Forward	ATCACCATCCGGAGGAAAG
	Reverse	TGCGGTTCTGAAACCAGATT
SOX2	Forward	TTGCTGCCTCTTTAAGACTAGGA
	Reverse	TAAGCCTGGGGCTCAAACT
SOX1	Forward	GAAATAGCCAATGCCAGGTG
	Reverse	CCGTGAATACGATGAGTGTTACC
OLIG2	Forward	AGCTCCTCAAATCGCATCC
	Reverse	ATAGTCGTCGCAGCTTTCG
NKX2.2	Forward	GCTGACCAACACAAAGACGG
	Reverse	GTTCTCTTCCTCCGGACCTT
HB9	Forward	TTACCTGACTTATGAAACTTGAAACC
	Reverse	CCCAGAGACGTAAGCATAAACC
ISL1	Forward	ACGGTGGCTTACAGGCTAAC
	Reverse	ATTAGAGCCCGGTCCTCCTT
LHX3	Forward	AAGTTCGGGACTGGAGAGTG
	Reverse	AGAGCCTTGAGGATGAAGCG
FOXP1	Forward	CAACGTGCCCATTTCGTCAG
	Reverse	GCGTCGGAAGTAAGCAAACA
MAP2	Forward	CAACGGAGAGCTGACCTCA
	Reverse	CTACAGCCTCAGCAGTGACTA
ChAT	Forward	GGACAACATCAGATCGGCCA
	Reverse	ACTGTGTATGCAGTCTGGGC
VAChT	Forward	CTTGTTATCGTGTGCGTGGC
	Reverse	GCGATGTAGTCGGGCACTAT
HOXC8	Forward	TCCCAGCCTCATGTTTCC

	Reverse	TGATACCGGCTGTAAGTTTGC
HOXA9	Forward	CAGGGTCTGGTGTTTTGTATAGGG
	Reverse	ACGCTTGACACTCACACTTTGTCC
HOXC9	Forward	TCCTAGCGTCCAGGTTTCC
	Reverse	GCTACAGTCCGGCACCAA
HOXC10	Forward	AGGAGAGGGCCAAAGCTG
	Reverse	AGCCAATTTCCTGTGGTGTT
HOXC13	Forward	TCTCCCTTCCCAGACGTG
	Reverse	CCGGCGCTTCTCTTTGGT
GAPDH	Forward	GCCCAATACGACCAAATCC
	Reverse	AGCCACATCGCTCAGACAC

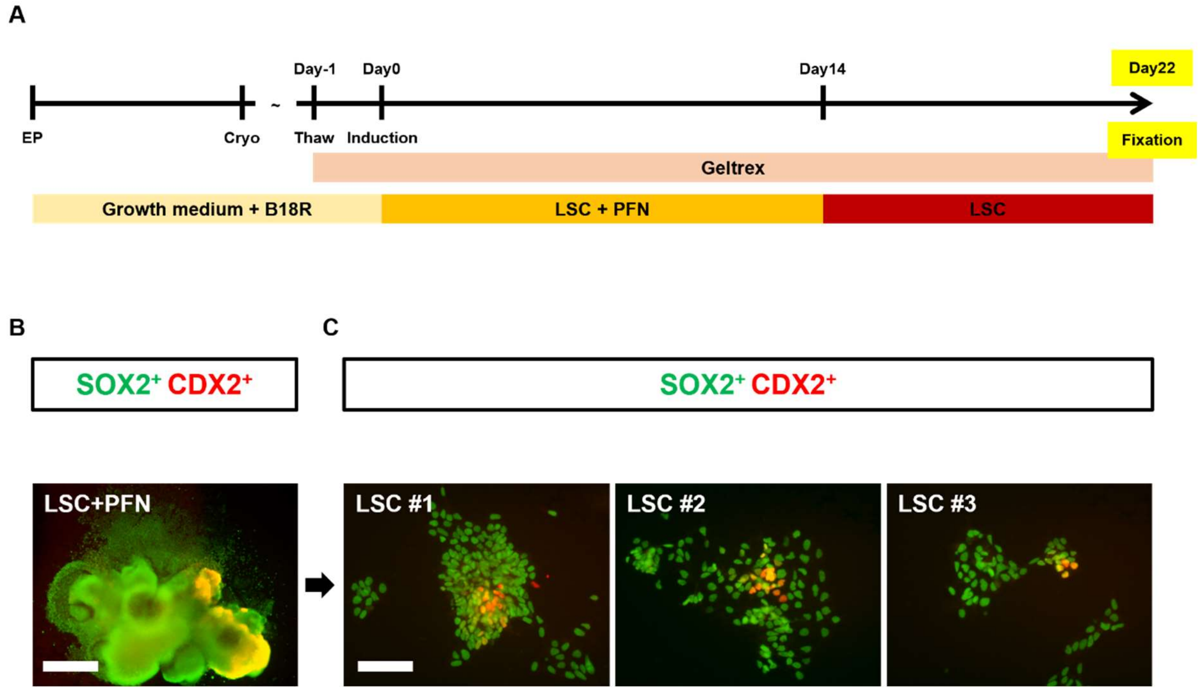

Supplementary Figure S1. Expression of posterior PNP-associated markers in human urine-derived iNSCs (A) Schematic of the time course of the process used to directly convert HUCs into iNSCs. **(B)** Immunofluorescence showing co-expression of the PNP marker CDX2 and the NSC marker SOX2 in an iNSC colony. **(C)** Co-expression of CDX2 and SOX2 in expanded iNSC subcultures following colony-derived passaging. Scale bars, 1mm **(B)**, 200 µm **(C)**

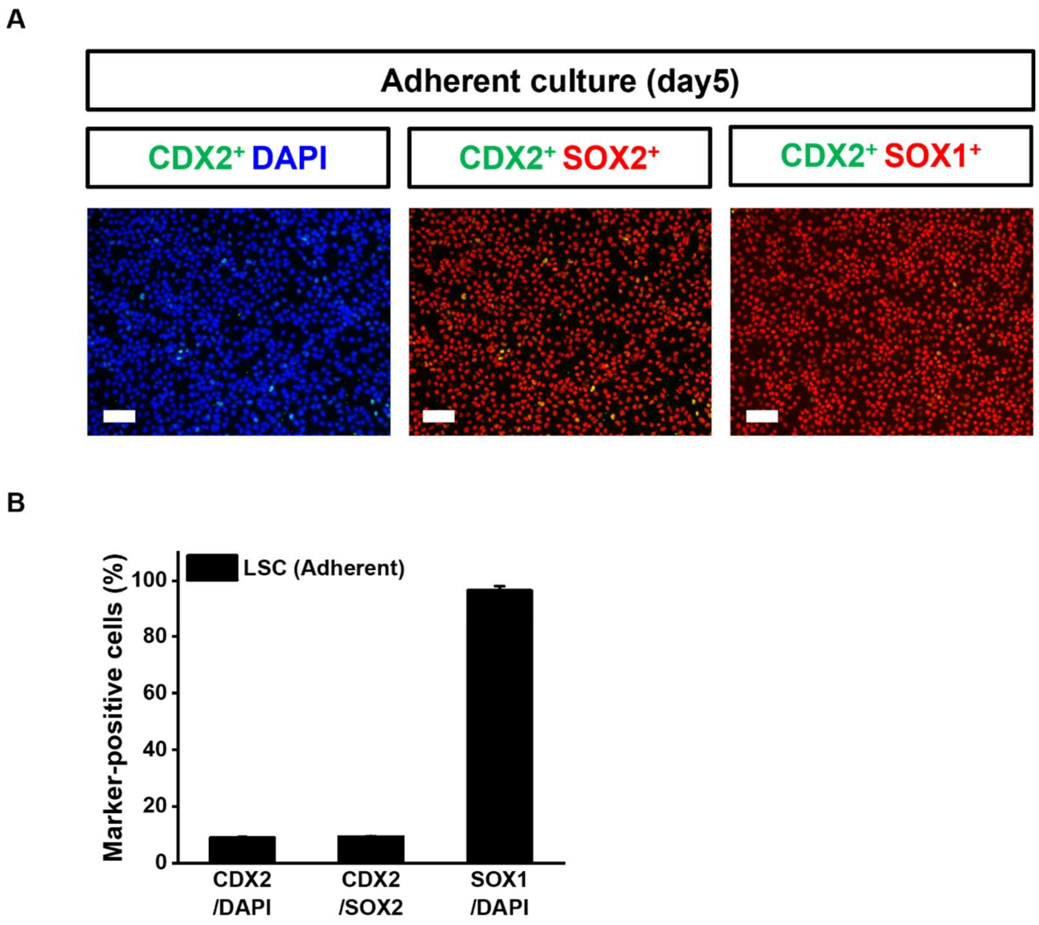

Supplementary Figure S2. Induction of a adherent posterior PNP H9 hPSC under LSC condition. (A) Cells were differentiated as adherent culture under LSC condition. Cells were stained for CDX2 and SOX2, with nuclei counterstained with DAPI. **(B)** Quantification of CDX2-positive and CDX2/SOX2-positive cells expressed as percentage of total DAPI-positive cells. Quantification was performed using ImageJ based on immunofluorescence images obtained from independent differentiation experiments (N = 3). Scale bars, 200 μm **(A)**

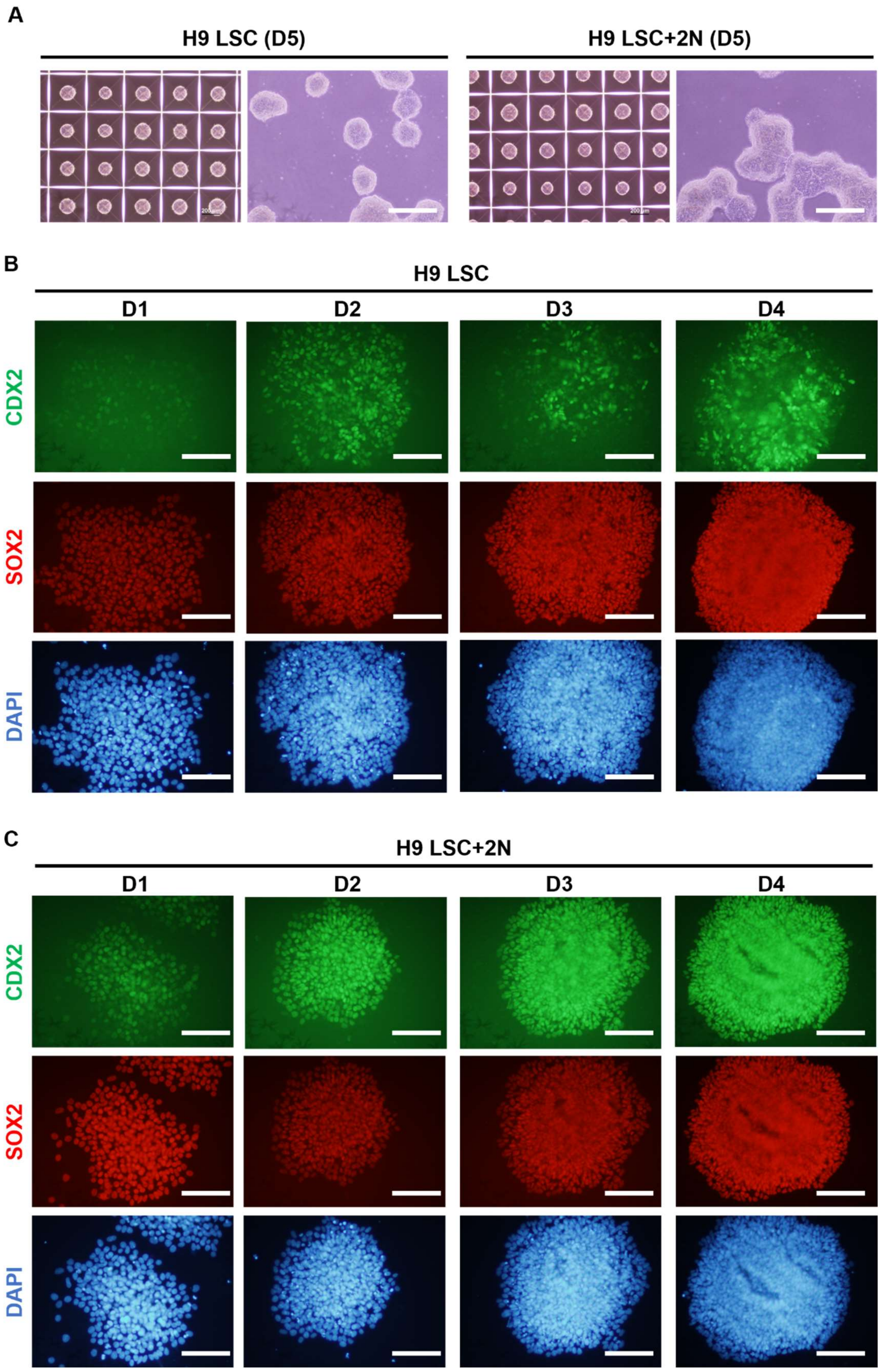
A
H9 LSC (D5)
H9 LSC+2N (D5)
B
H9 LSC
D1
D2
D3
D4
CDX2
SOX2
DAPI
C
H9 LSC+2N
D1
D2
D3
D4
CDX2
SOX2
DAPI

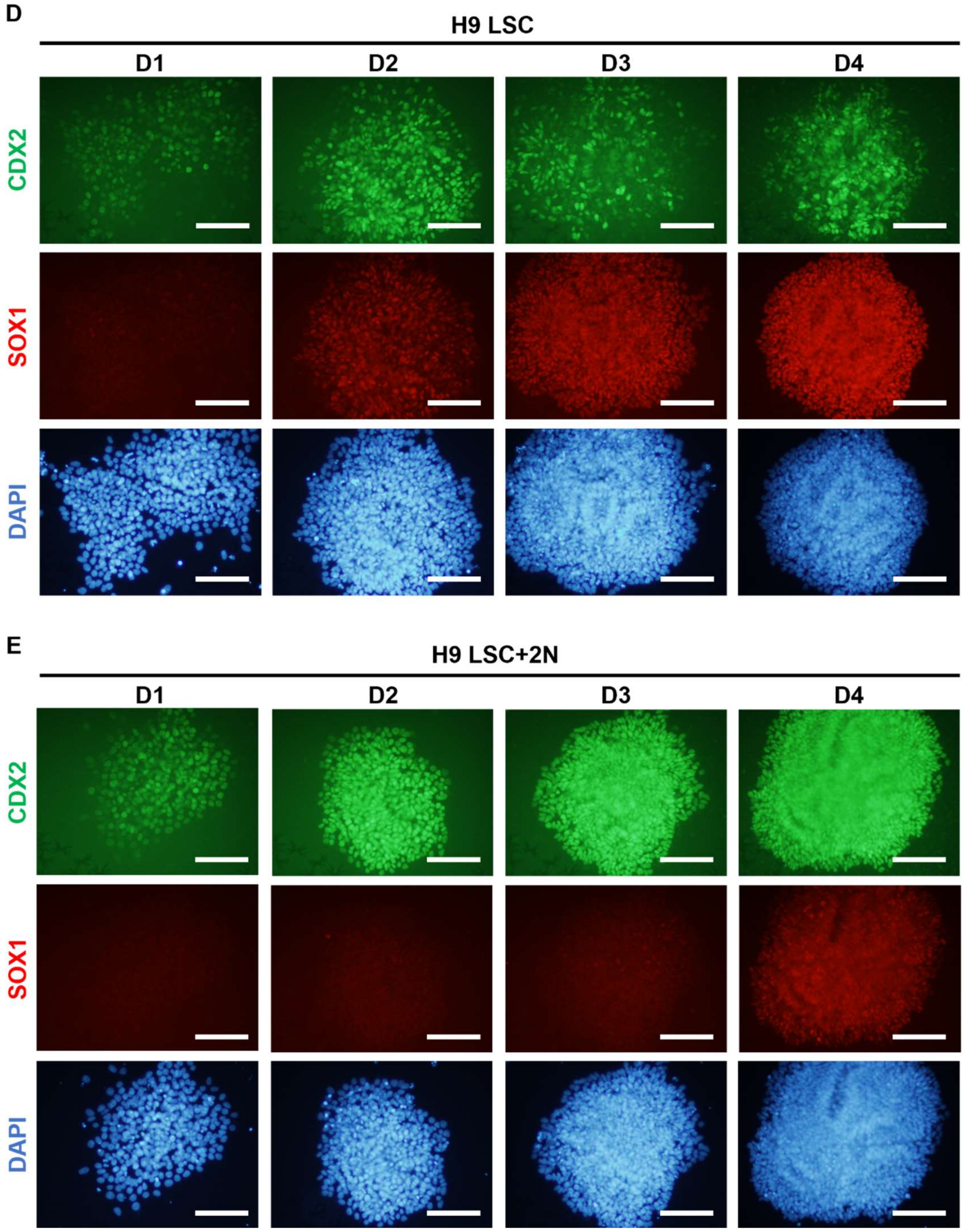

Supplementary Figure S3. Morphology and temporal expression of CDX2 and SOX markers in H9 hESCs under LSC and LSC+2N conditions (A) Morphology of H9 human embryonic stem cells (ESCs) at day 5 following induction under LSC or LSC+2N conditions. **(B–C)** Immunofluorescence staining of CDX2 and SOX2 during daily differentiation under LSC and LSC+2N conditions. **(D–E)** Immunofluorescence analysis of CDX2 and SOX1 expression over a daily time course under LSC and LSC+2N conditions. Scale bars, 1mm(A), 200 μm (B–E**)**

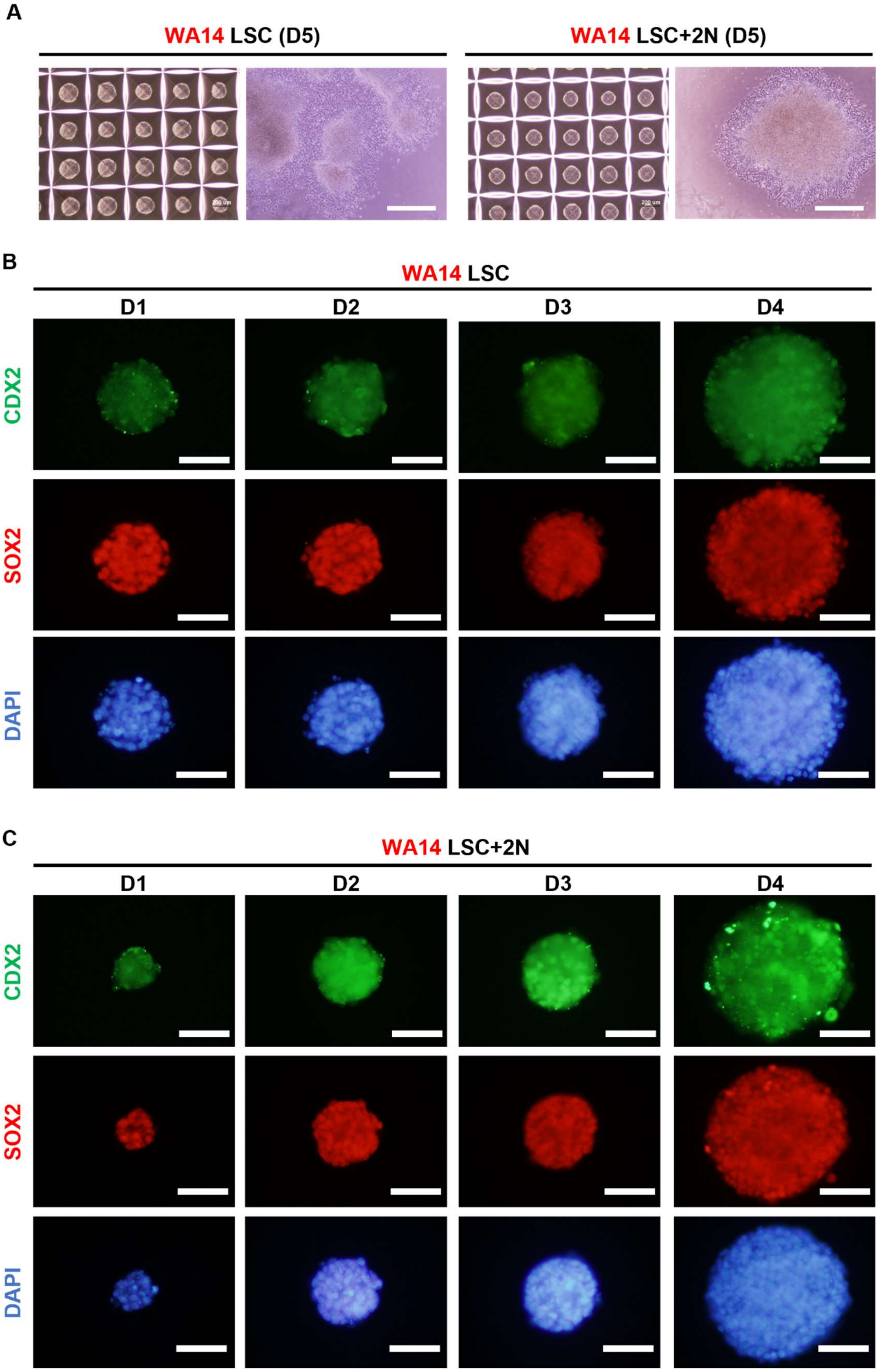
A
WA14 LSC (D5)
WA14 LSC+2N (D5)
B
WA14 LSC
D1
D2
D3
D4
CDX2
SOX2
DAPI
C
WA14 LSC+2N
D1
D2
D3
D4
CDX2
SOX2
DAPI

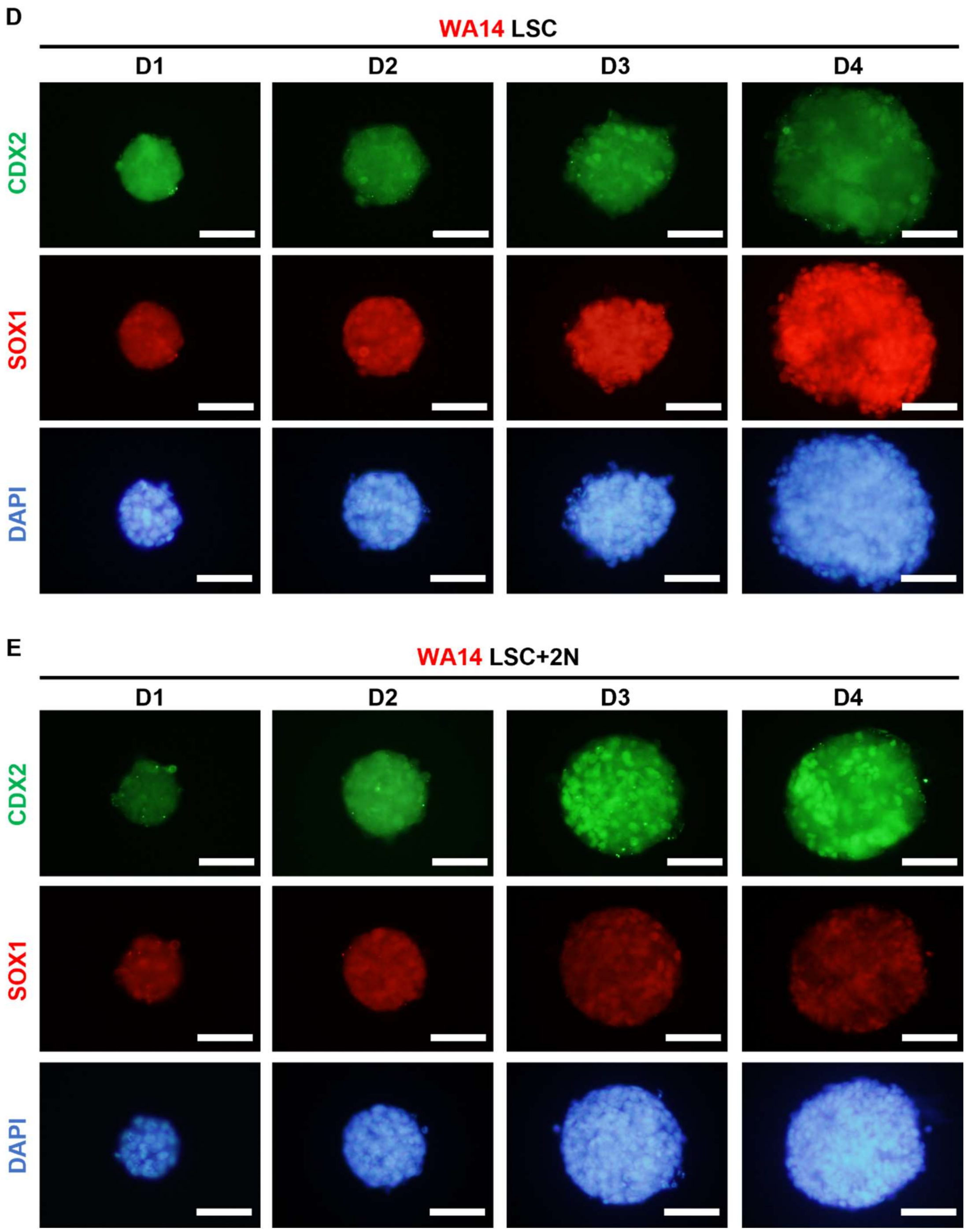

Supplementary Figure S4. Morphology and temporal expression of CDX2 and SOX markers in WA14 hESCs under LSC and LSC+2N conditions (A) Morphology of WA14 ESCs at day 5 following induction under LSC or LSC+2N conditions. **(B–C)** Immunofluorescence staining of CDX2 and SOX2 during daily differentiation under LSC and LSC+2N conditions. **(D–E)** Immunofluorescence analysis of CDX2 and SOX1 expression over a daily time course under LSC and LSC+2N conditions. Scale bars, 1mm(A), 200 μm (B–E**)**

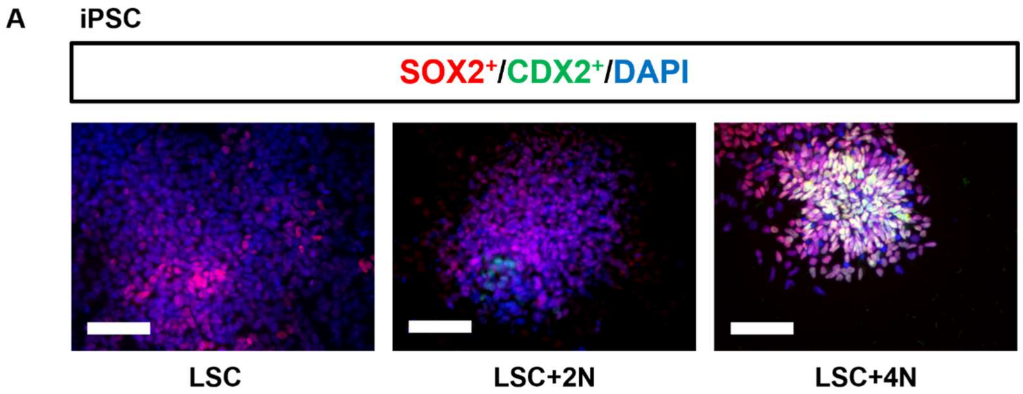

Supplementary Figure S5. Expression of CDX2 and SOX2 markers in iPSCs under LSC, LSC+2N and LSC+ 4N conditions (A) Representative immunofluorescence images of iPSC derived spheres at day 5 of differentiation cultured under LSC, LSC+2N, and LSC+4N conditions. Nuclei were counterstained with DAPI. Scale bars, 200 μm

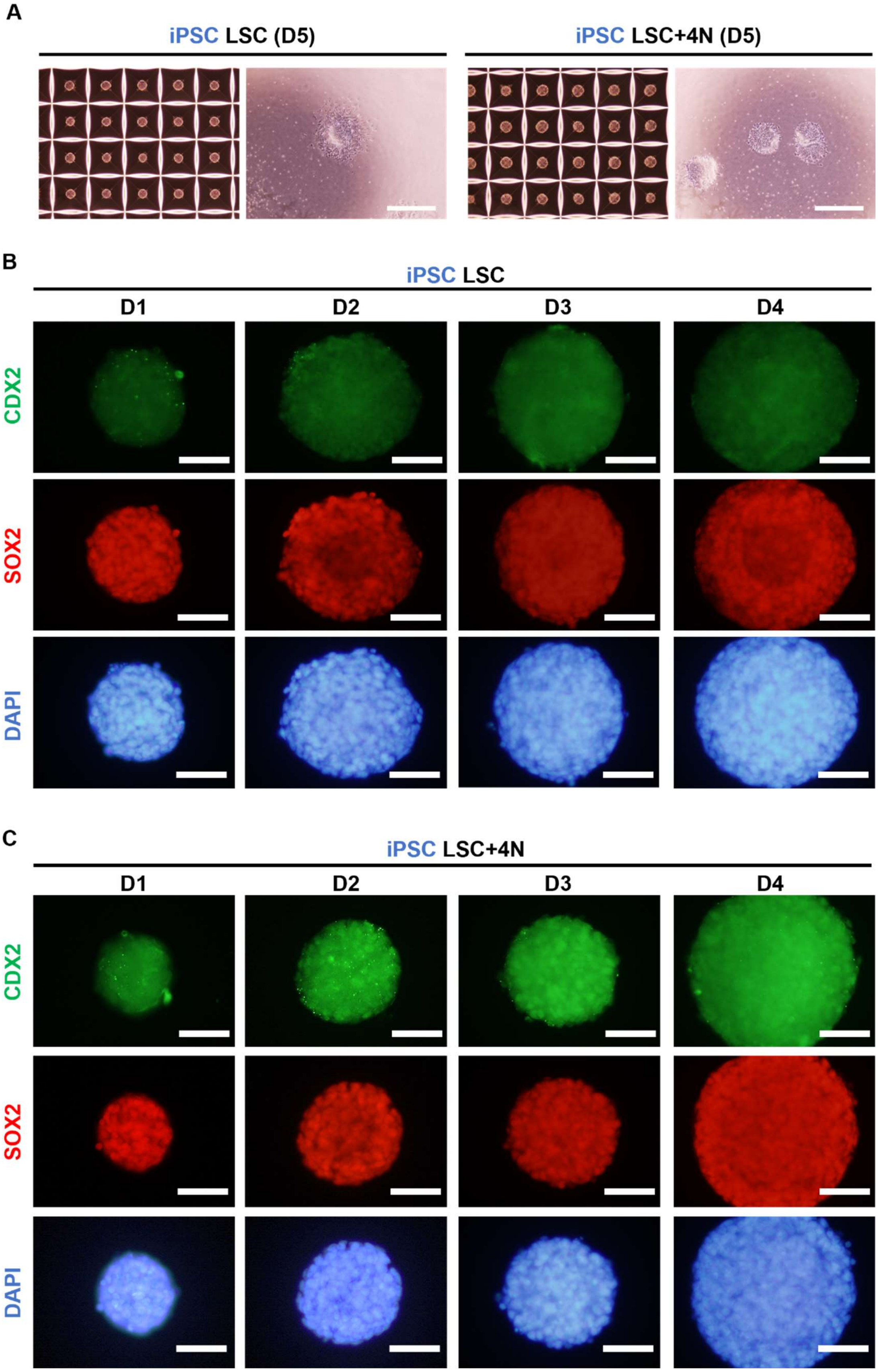
A
iPSC LSC (D5)
iPSC LSC+4N (D5)
B
iPSC LSC
D1
D2
D3
D4
CDX2
SOX2
DAPI
C
iPSC LSC+4N
D1
D2
D3
D4
CDX2
SOX2
DAPI

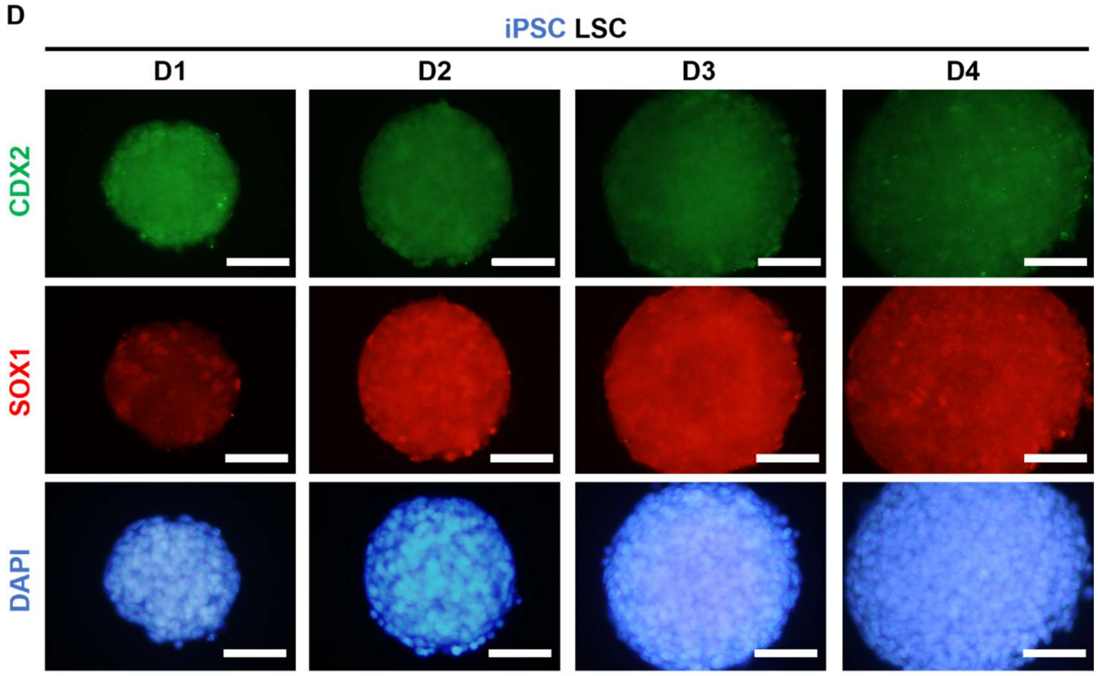

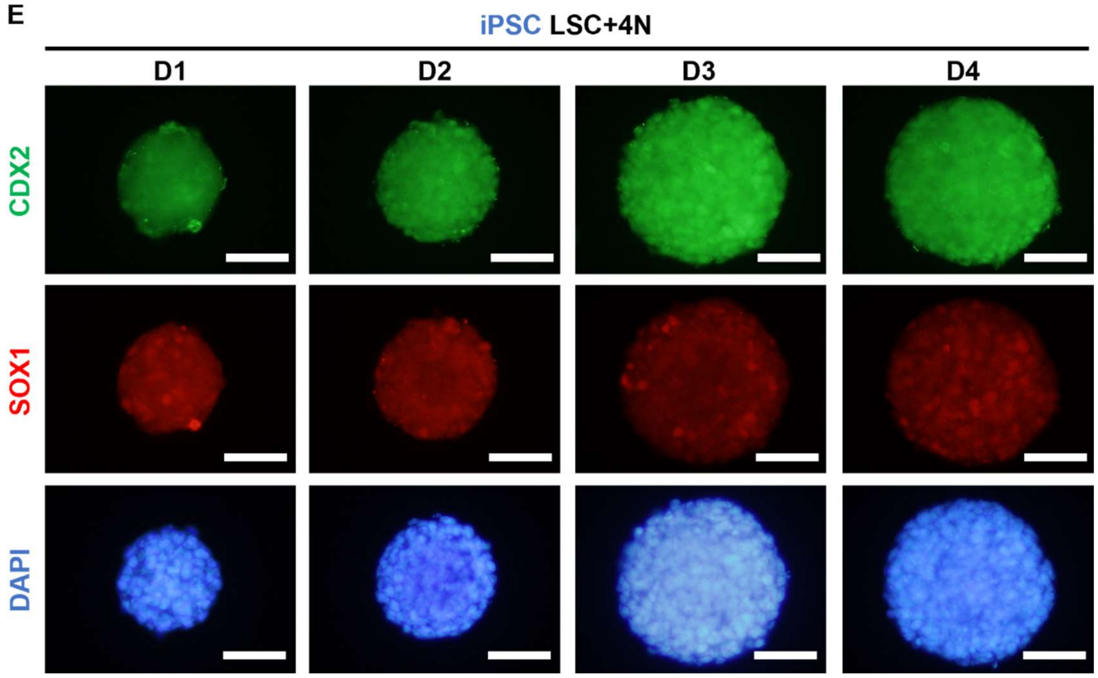

Supplementary Figure S6. Morphology and temporal expression of CDX2 and SOX markers in iPSCs under LSC and LSC+4N conditions (A) Morphology of IPSCs ESCs at day 5 following induction under LSC or LSC+2N conditions. **(B–C)** Immunofluorescence staining of CDX2 and SOX2 during daily differentiation under LSC and LSC+4N conditions. **(D–E)** Immunofluorescence analysis of CDX2 and SOX1 expression over a daily time course under LSC and LSC+4N conditions. Scale bars, 1mm(A), 200 μm (B–E**)**

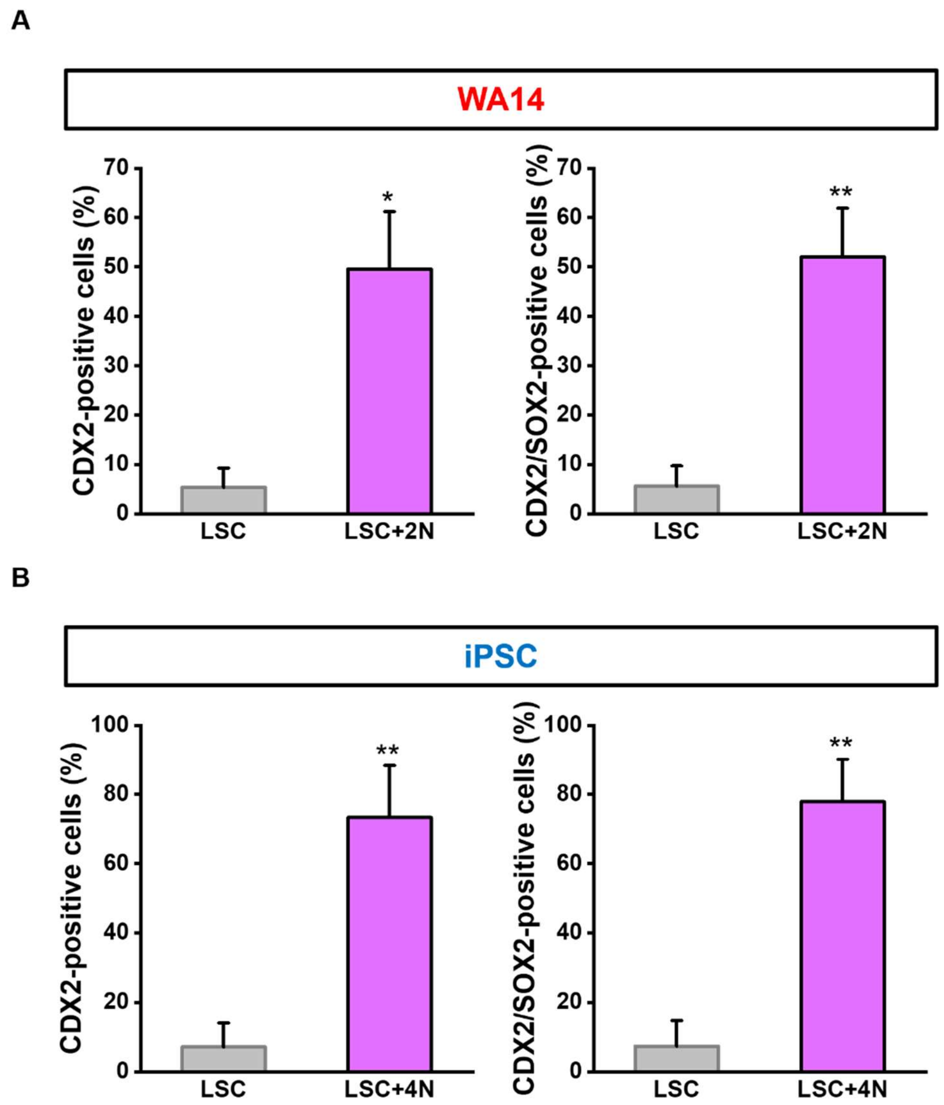

Supplementary Figure S7. Quantification of $CDX2^+$ and $CDX2^+/SOX2^+$ positive cells in WA14 ESC and iPSCs under LSC and LSC+2N,4N conditions. (A) Quantification of CDX2-positive and CDX2/SOX2-positive cells expressed as percentages of total DAPI-positive cells in WA14 under LSC, LSC+2N conditions. (B) Quantification of CDX2-positive and CDX2/SOX2-positive cells expressed as percentages of total DAPI-positive cells in iPSCs under LSC, LSC+2N conditions. Quantification was performed using ImageJ based on confocal images obtained from independent differentiation experiments (N = 3). Data are presented as mean ± SD. Statistical significance was determined using one-way ANOVA followed by post hoc tests. $*p < 0.05$, $**p < 0.01$.

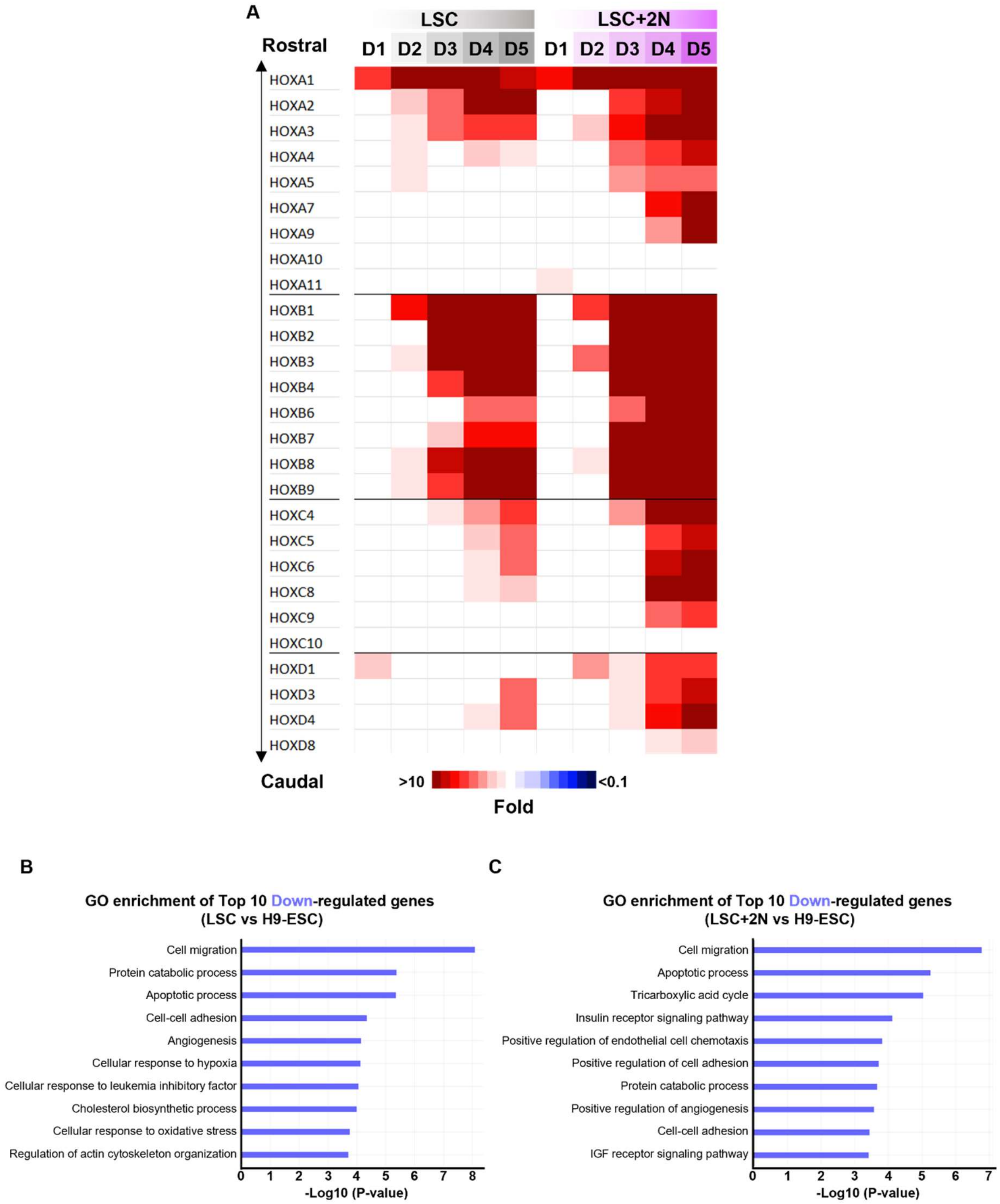

Supplementary Figure S8. Posterior HOX gene expression and downregulated biological process enrichment under LSC and LSC+2N conditions. (A) Heatmap of selected marker genes across D1–D5 under LSC and LSC+2N conditions, including posterior *Hox* genes (fold change relative to H9-ESC; color scale as shown). GO Biological Process enrichment of the top 10 downregulated genes in LSC vs H9-ESC **(B)** and LSC+2N vs H9-ESC **(C)**, plotted as −log10(p value).

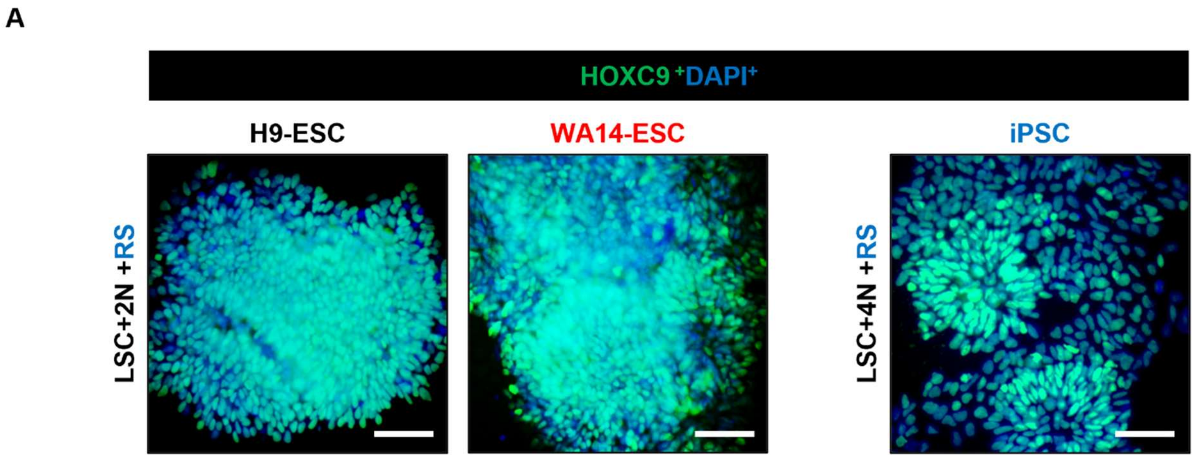

Supplementary Figure S9. HOXC9 expression in multiple pluripotent stem cell–derived cultures following RA and SAG treatment. (A) Representative immunofluorescence images showing induction of the posterior neural tube marker HOXC9 in differentiated cultures derived from H9, WA14 ESCs, and iPSCs following RA and SAG treatment. Nuclei were counterstained with DAPI. Scale bars, 200 μm

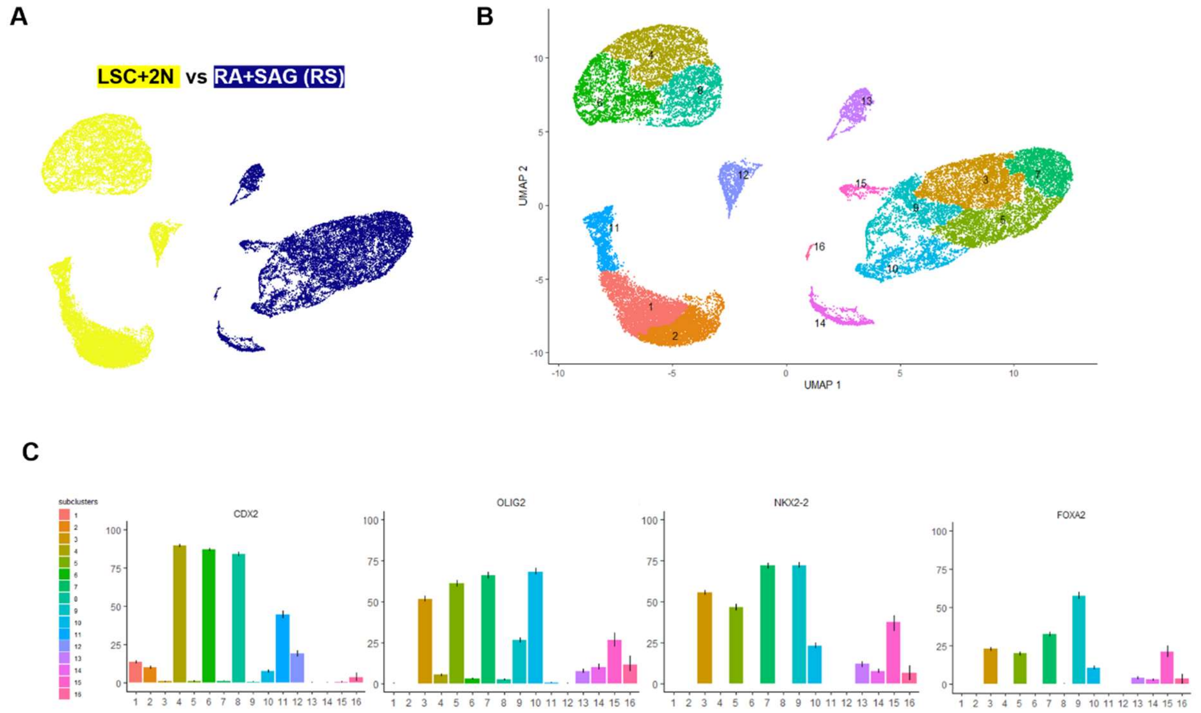

Supplementary Figure S10. Marker expression analysis across transcriptionally distinct clusters. (A) UMAP visualization of cells differentiated under LSC+2N and RA+SAG (RS) conditions. **(B)** UMAP analysis was performed to resolve transcriptionally distinct cell populations. **(C)** Expression profiles of representative markers associated with pre-neural progenitors, motor neuron progenitors, and ventral progenitors and floor plate state showed differential enrichment across individual cluster.

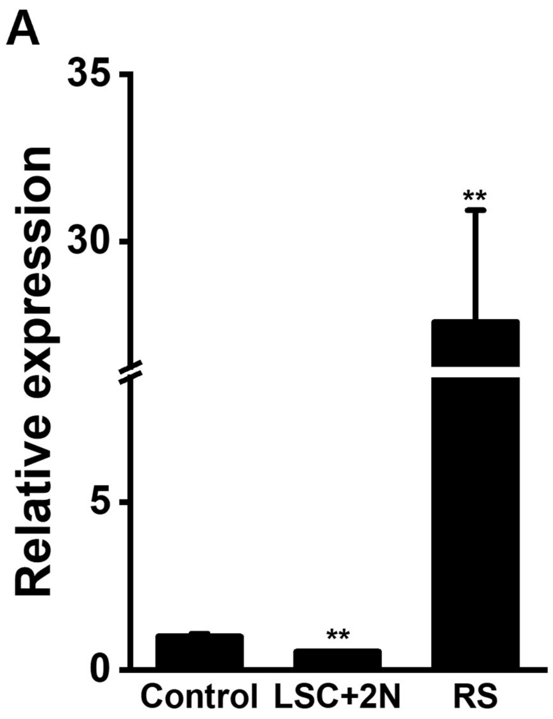

Supplementary Figure S11. HES5 expression during posterior PNP induction and neural tube patterning. (A) RT–qPCR analysis of HES5 expression during differentiation under the indicated culture conditions. HES5 expression was evaluated in human pluripotent stem cells (ESC), LSC+2N at day 5, and RS-treated cultures. HES5 expression remained low during the LSC+2N-mediated posterior pre-neural progenitor (PNP) stage but was markedly upregulated following RS-mediated ventral neural tube patterning, consistent with the transition from a posterior pre-neural state to a patterned neural tube identity. Data are presented as mean ± SD from independent experiments (N = 4). Statistical significance was determined using one-way ANOVA followed by appropriate post hoc tests. **$p < 0.01$

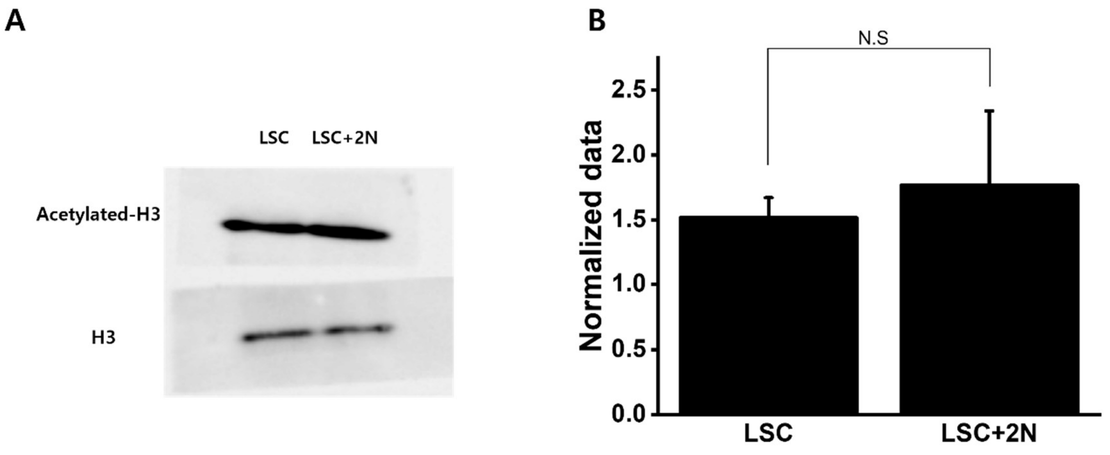

Supplementary Figure S12. Analysis of global H3K9 acetylation during posterior PNP induction.

(A) Western blot analysis of acetylated histone H3 at lysine 9 (H3K9ac) and total histone H3 in LSC- and LSC+2N-treated cultures at day 5. (B) H3K9ac levels were normalized to total histone H3. Although the LSC+2N group showed a modest increase in the H3K9ac/H3 ratio compared with the LSC group, the difference was not statistically significant. Data are presented as mean ± SD from independent experiments (N = 2). Statistical significance was determined using an unpaired two-tailed Student's *t*-test. ns, not significant.
